# Solution-State Inter-Copper Distribution of Redox Partner-Linked Copper Nitrite Reductases: A Pulsed Electron–Electron Double Resonance Spectroscopy Study

**DOI:** 10.1021/acs.jpclett.2c01584

**Published:** 2022-07-22

**Authors:** Tobias M. Hedison, Andreea I. Iorgu, Donato Calabrese, Derren J. Heyes, Muralidharan Shanmugam, Nigel S. Scrutton

**Affiliations:** † Manchester Institute of Biotechnology and Department of Chemistry, University of Manchester, 131 Princess Street, Manchester M1 7DN, U.K.; ‡ EPSRC/BBSRC funded Future Biomanufacturing Research Hub, University of Manchester, 131 Princess Street, Manchester M1 7DN, U.K.

## Abstract

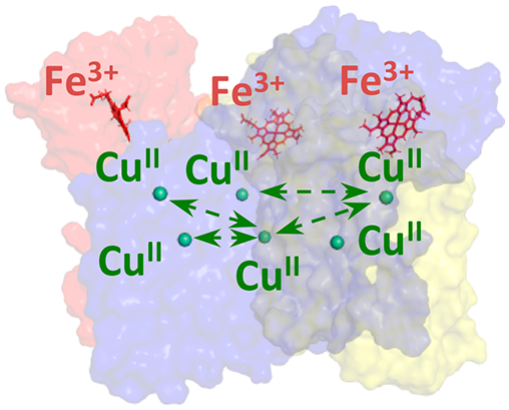

Copper nitrite reductases (CuNiRs) catalyze the reduction of nitrite to form nitric oxide. In recent years, new classes of redox partner linked CuNiRs have been isolated and characterized by crystallographic techniques. Solution-state biophysical studies have shed light on the complex catalytic mechanisms of these enzymes and implied that protein dynamics may play a role in CuNiR catalysis. To investigate the structural, dynamical, and functional relationship of these CuNiRs, we have used protein reverse engineering and pulsed electron–electron double resonance (PELDOR) spectroscopy to determine their solution-state inter-copper distributions. Data show the multidimensional conformational landscape of this family of enzymes and the role of tethering in catalysis. The importance of combining high-resolution crystallographic techniques and low-resolution solution-state approaches in determining the structures and mechanisms of metalloenzymes is emphasized by our approach.

The wealth of information provided by high-resolution protein X-ray crystallographic structures has been essential in the determination of protein function and mechanism for many decades. However, crystallographic data often provide a snapshot of protein conformational ensembles, and other complementary techniques are frequently used to access and gain knowledge of the broader dynamic-structural landscape of proteins.^1^ One method that has been used to study the conformational space explored by a protein is electron paramagnetic resonance (EPR) spectroscopy, which due to the development of pulsed EPR methods has experienced a resurgence in the study of biological macromolecules.^2,3^ Pulsed electron–electron double resonance (PELDOR) spectroscopy is an advanced EPR technique that can be used to measure the nanometer distance (2–6 nm for the sample in the deuterated buffer, 2–10 nm for the perdeuterated samples) between pairs of unpaired electrons (e.g., nitroxide spin-labels, tyrosyl radicals, and flavin semiquinones) or open shell transition-metal centers (e.g., Cu^II^ ions or ferric heme species) found within or bound to proteins.^4−9^ Cu^II^ ions are frequently used as spin probes in pulse dipolar spectroscopy (PDS) as they are omnipresent in nature.^10^ However, because of their large *g* and “hyperfine” anisotropies, challenges with “orientation selectivity” are often encountered when conducting PELDOR spectroscopy on Cu-containing proteins.^11−13^ Previous studies have shown that “orientation selection” can be minimized by conducting PELDOR at *g*_perp_, where molecules with a wide range of orientations are superimposed.^11−13^ In recent years, PELDOR has proven to be a useful low-resolution structural technique and for example has been used to probe the substrate-coupled conformational cycle of the mouse ABC efflux transporter, P-glycoprotein,^14^ as well as the solution structure of the discoidal high-density lipoprotein (rdHDL)^15^ and particulate methane monooxygenase (pMMO).^16^ The recent PELDOR community paper (and selected PELDOR references therein), which was led by Schiemann et al., elegantly sets up the standards for reporting PELDOR data on the nitroxide-labeled biomolecules and analogues systems.^17^

Copper-containing nitrite reductases (CuNiRs) catalyze the reduction of nitrite to form nitric oxide, a key step in the nitrogen cycle.^18^ Two-domain CuNiRs have been extensively studied by biochemical methods.^19−21^ Irrespective of origin, two-domain CuNiRs are homotrimeric.^22^ In each CuNiR monomer, there are two cupredoxin domains, both housing a copper ion center—either a type I (T1Cu) or a type II Cu (T2Cu).^21^ Two histidines, one cysteine and an axial methionine residue, coordinate the T1Cu ion in nitrite reductases. The T2Cu center of CuNiRs is coordinated in a tetrahedral geometry by three histidine residues and a water/nitrite/nitric oxide molecule.^21^ Mechanistic studies show that partner proteins (azurin or cytochrome *c*) deliver electrons to the T1Cu site of CuNiRs. Electrons then transfer along the characteristic CuNiR Cys-His bridge to the T2Cu site, where nitrite is reduced to nitric oxide. Because of the presence of the six copper ion centers (Figure 1), which are EPR-active in their Cu^II^ oxidation state, CuNiRs are highly amenable to EPR spectroscopy. PELDOR spectroscopy has previously been used by van Wonderen et al. to map the distances between the copper centers in the two-domain CuNiR from the Gram-negative bacterium *Alcaligenes xylosoxidans* (*Ax*NiR).^3^

**Figure 1:**
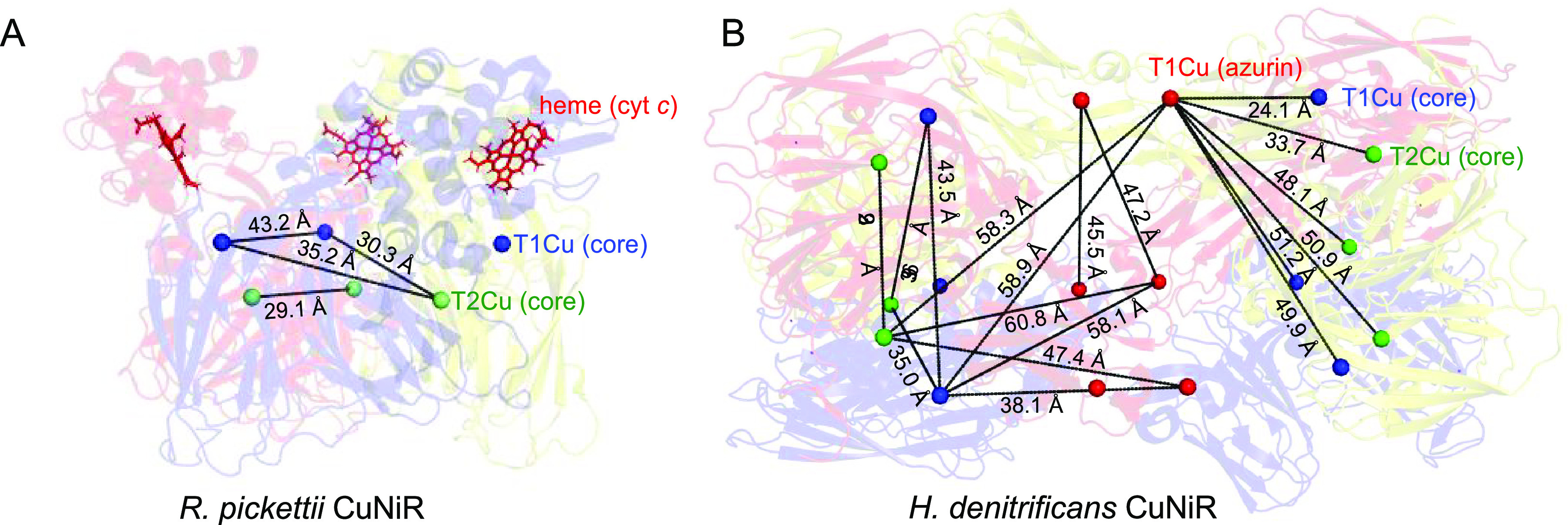
X-ray crystallographic structures of three-domain, redox partner-linked copper-containing nitrite reductases along with the intercenter distances that in principle can be measured by using PELDOR. (A) X-ray Structure of *Ralstonia pickettii* copper nitrite reductase (*Rp*NiR; PDB ID: 3ZIY; C-terminal cytochrome *c*-tethered CuNiR). (B) X-ray Structure of *Hyphomicrobium denitrificans* CuNiR copper nitrite reductase (*Hd*NiR; PDB ID: 2DV6; N-terminal Az-tethered CuNiR). In the structures, the inter-copper distances that can be measured by the PELDOR technique are shown by black lines. Each of the monomeric units in *Rp*NiR is represented as a red, yellow, and blue cartoon. The T1Cu and T2Cu centers within the core region are shown as blue and green spheres. The T1Cu center in the fused cupredoxin domain of *Hd*NiR is shown as a red sphere, and the heme cofactor is presented as red sticks. As *Hd*NiR is a dimer of trimers, each of the monomers within the two separate trimeric halves is represented as a red, yellow, and blue cartoon.

Over the past decade, a new class of CuNiRs has been identified, isolated, and characterized by biophysical and crystallographic methods.^22−26^ These partner protein-linked CuNiRs contain the same “core” CuNiR structure but exist with either N- or C-terminal azurin or cytochrome “partner protein” domains (Figure 1).^25,26^ There is evidence from small-angle X-ray scattering (SAXS) and laser flash photolysis studies that protein domain dynamics play a role in heme to T1Cu electron transfer in C-terminal cytochrome *c*-fused CuNiRs.^24^ Likewise, the unexpected >15 Å T1Cu–T1Cu distance between the T1Cu of the linked azurin domain and the “core” T1Cu in the crystallographic structure of the three-domain azurin-tethered CuNiR further supports a role of dynamics in the redox partner-linked CuNiRs.^26^ These dynamic properties, which we recently reported to be essential for catalysis in two-domain CuNiRs,^27^ can be “hidden” in the “static” crystallographic structures. Complementary low-resolution solution-based structural techniques can often provide a more complete picture of enzyme structure and function. Therefore, to gain additional structural and dynamical insight into redox-partner fused CuNiRs, we have performed PELDOR spectroscopy on the three-domain cytochrome *c*-fused CuNiR from *Ralstonia pickettii* (*Rp*NiR) and the three-domain, azurin*-*fused *Hyphomicrobium denitrificans* CuNiR (*Hd*NiR) to determine the inter-copper distribution in these proteins.

On the basis of X-ray crystallographic structures of the three-domain *R. pickettii* and *H. denitrificans* CuNiR enzymes, there are four discrete sets of Cu^II^–Cu^II^ distances within the “core” region of both *Rp*NiR and *Hd*NiR that theoretically can be measured by using PELDOR spectroscopy (Figure 1). These are a set of three intermonomer T1–T1Cu distances, two sets of three intermonomer T1–T2Cu distances, and a set of three intermonomer T2–T2Cu distances.^4^ The intramonomer T1–T2Cu distance (∼12.6 Å) is too short to be accurately measured by using PELDOR spectroscopy.^4^ In the full-length *Hd*NiR protein, there are 12 additional discrete sets of distances that are within the range to be accurately measured by PELDOR spectroscopy (Figure 1B).^4^ Similarly, in the full-length *Rp*NiR, there are six additional sets of distances (heme–heme and heme–Cu^II^) that can be theoretically detected by using PELDOR spectroscopy (Figure 1A). Our work is based on that previously conducted by van Wonderen in which the iDEER approach was used.^3^ As these experiments were previously conducted on the highly similar *Ax*NiR, they could be used to guide interpretation of our results on the three-domain *Rp*NiR and *Hd*NiR. As the core structures of all CuNiRs are similar, we interpreted our results based on the iDEER results from this work.^3^ Therefore, to begin our study and to reduce the complexity from the multitude of prospective distances recorded in the full-length three-domain CuNiRs, we isolated the *Rp*NiR and *Hd*NiR “core” regions (the two-domain portion of the CuNiRs) by reverse engineering and removal of the C- and N-terminal cytochrome^24^ and azurin domains, respectively (Figures 2, S1, and S2). For simplicity, these “core” portions of the three-domain NiRs were recombinantly expressed with N-terminal His-tags and subsequently purified by nickel affinity chromatography. As expected (and similar to all other two-domain NiRs),^22^ gel filtration chromatography of the His-tag cleaved *Rp*NiR and *Hd*NiR “cores” showed these proteins were trimeric. Continuous wave EPR spectra of both truncated constructs show the resolved ^63,65^Cu hyperfine features along the *g*_II_ region, arising from the interaction of an electron spin (*S* = 1/2) of Cu^II^ with the nuclear spin (*I* = 3/2) of ^63,65^Cu nuclei [*g*_II_ = 2.214, *A*_II_(^63,65^Cu; T1Cu) = 107 MHz and *g*_II_ = 2.314, *A*_II_(^63,65^Cu; T2Cu) = 402 MHz for the *Rp*NiR “core”^27,28^ and *g*_II_ = 2.244, A_II_(^63,65^Cu; T1Cu) = 170 MHz and *g*_II_ = 2.255, *A*_II_(^63,65^Cu; T2Cu) = 458 MHz for the *Hd*NiR “core”] (Figures S3 and S4).^27,29^ The simulated spectra and the spin-Hamiltonian parameters for the *Hd*NiR “core” are given in Figures S5 and S6. The T2Cu sites of both “core” portions were shown to be ∼30% occupied (refer to Figures S3–S8 for more details about the T2Cu occupancy), a property of these enzymes that was identified in our previously published work.^30^ Attempts to increase T2Cu occupancy with further copper supplementation were unsuccessful. Therefore, it should be noted that in our PELDOR spectroscopic data dipolar modulations arising from the inter-copper distances associated with the T2Cu center are weaker than expected (Figure 2; see the Supporting Information for raw data).

**Figure 2:**
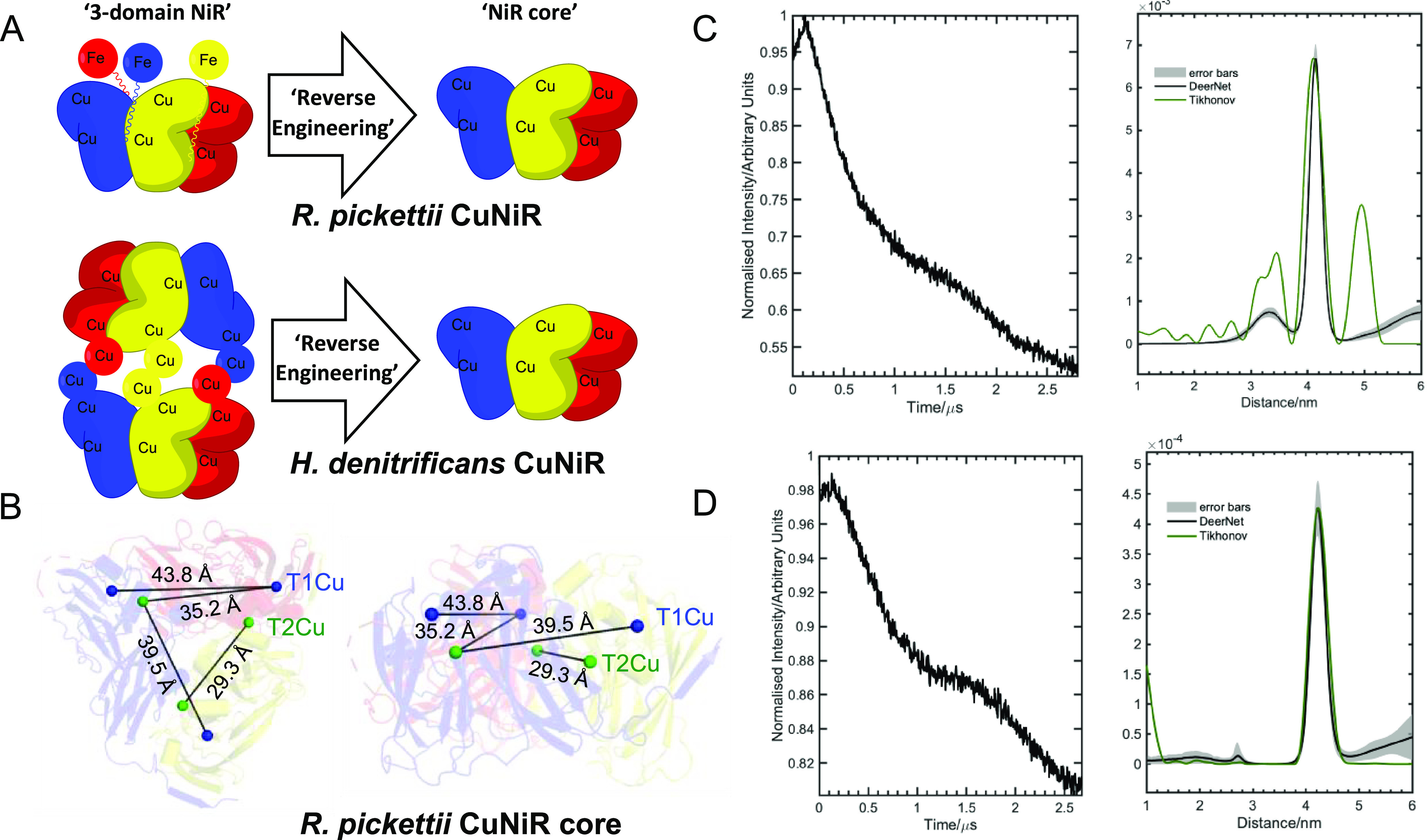
Solution-state inter-copper distribution of the deconstructed “core” regions from the three-domain redox-partner linked copper nitrite reductases. (A) Schematic showing the molecular architecture of the three-domain copper-containing nitrite reductases from *Ralstonia pickettii* (top) and *Hyphomicrobium denitrificans* (bottom) and the design of “core” constructs used to probe the inter-copper “solution state” of these proteins. (B) X-ray structure of the *Ralstonia pickettii* copper-containing nitrite reductase (PDB ID: 6QPU) showing the inter-copper distance distribution of these proteins. PELDOR time traces (left-hand panels of parts C and D) and the comparisons of PELDOR distance distribution outputs using the Tikhonov regularization (green traces; right-hand panels of parts C and D) and DEERNet analysis (black traces; right-hand panels of parts C and D) methods for the deconstructed “core” portion of the three-domain (C) *Ralstonia pickettii* and (D) *Hyphomicrobium denitrificans* copper-containing nitrite reductase. PELDOR distance distribution validation is presented here by using the gray error bars. The PELDOR distance distribution analysis by the Tikhonov method, including the validation for the “core” and full length *Rp*-NiR and *Hd*-NiR proteins, is provided in Figures S10, S12, S14, and S21. Each of the monomeric units in the *Ralstonia pickettii* and *Hyphomicrobium denitrificans* copper-containing nitrite reductases shown in (A) and (B) are represented as a red, yellow, or blue cartoon. The T1 and T2Cu centers in the *Rp*NiR core structure are shown as blue and green spheres, respectively.

The background-subtracted X-band PELDOR time traces are provided in Figures 2C and 2D (left-hand panels) and also in Figures S9 and S11. The corresponding inter-copper distance distributions for the oxidized *Rp*NiR and *Hd*NiR “core” portions measured at 10 K are shown in Figure 2 (the continuous-wave EPR, two-pulse field-swept echo-detected EPR spectra, raw PELDOR data, and validation of distance distribution for the *Rp*NiR and *Hd*NiR “core” are shown in Figures S3–S12). The PELDOR data were analyzed by using DeerAnalysis2022^31^ and DEERNet Spinach SVN Rev 5662.^32,33^ Analysis of the continuous-wave EPR spectra was performed by using the EasySpin toolbox (5.2.35) that is adapted for the Matlab program package.^34^ To minimize the effect of “orientation selectivity”, PELDOR data were collected at frequencies close to *g*_⊥_(3310–3350 G), where contributions from molecules with a wide range of orientations are overlapped.^3,35^ For analysis of these data sets, Tikhonov regularization and neural network (DEERNet) analysis are employed (see the description in the Supporting Information for more details).^3,31^

PELDOR measurements performed on the *Rp*NiR “core” protein show the presence of six discrete inter-copper distances at 22.8, 26.5, 31.6, 34.2, 40.8, and 47.8 Å. The major distance, recorded at 40.8 Å, is ∼2 times more intense than the next highest peak in our PELDOR analysis (Figure 2C). A similar result was previously observed by van Wonderen et al.^3^ when conducting PELDOR on the two-domain *Ax*NiR, and as stated in their work, it is highly likely that the distance measured at 40.8 Å for the *Rp*NiR “core” protein is a collection of the T1–T1 Cu distances (43.8 Å in the crystal structure; Figure 2B) and the longer of the two T1–T2Cu distances (39.5 Å in the crystal structure; Figures 2B, S9, and S10). The Tikhonov validation of 30 trials proved the validity of the five additional distances observed. However, the PELDOR trace analyzed by DEERNet (Figure S10) shows a predominant distance at 40.8 Å and a broad distance distribution from 30 to 36 Å. The additional distance distribution peaks, below 30 Å and above 50 Å, obtained in the Tikhonov method were not identified in the DEERNet analysis. This comparison suggests that the additional distance distribution obtained from the Tikhonov regularization method could arise from the errors in the background fitting and/or artifacts. However, we cannot rule out the possibilities that the five additional distances, which were detected by using Tikhonov regularization method, represent two major conformational states of the *Rp*NiR “core”. The two short distances (at 22.8 and 26.5 Å) represent two T2–T2Cu states, while the two distances at 31.6 and 34.2 Å arise from the shorter T1–T2Cu distance. The two “major” Cu^II^–Cu^II^ distances (at 40.8 and 47.8 Å) are attributed to two collections of the sum of the longer T1–T2Cu distance and the T1–T1Cu distance. We note that the observed modulation depth is 0.23–0.25 in both the “core” and the full-length *Rp*NiR proteins (Figures 2 and 3), so the multispin effects could be neglected in these measurements.^36,37^ Altogether, the PELDOR structural data recorded on the “core” portion of *Rp*NiR illustrate that the protein adopts at least two conformational states in solution. The occurrence of two predominant structures of the *Rp*NiR “core” protein seen in this PELDOR study could explain why biphasic inter-copper electron transfer kinetics are observed in our previously published flash photolysis measurements on the *Rp*NiR “core”.^24^ On the basis of this PELDOR work, it is possible that each of these two kinetic phases is attributed to one of these two distinct conformational states of the *Rp*NiR “core” protein.

**Figure 3:**
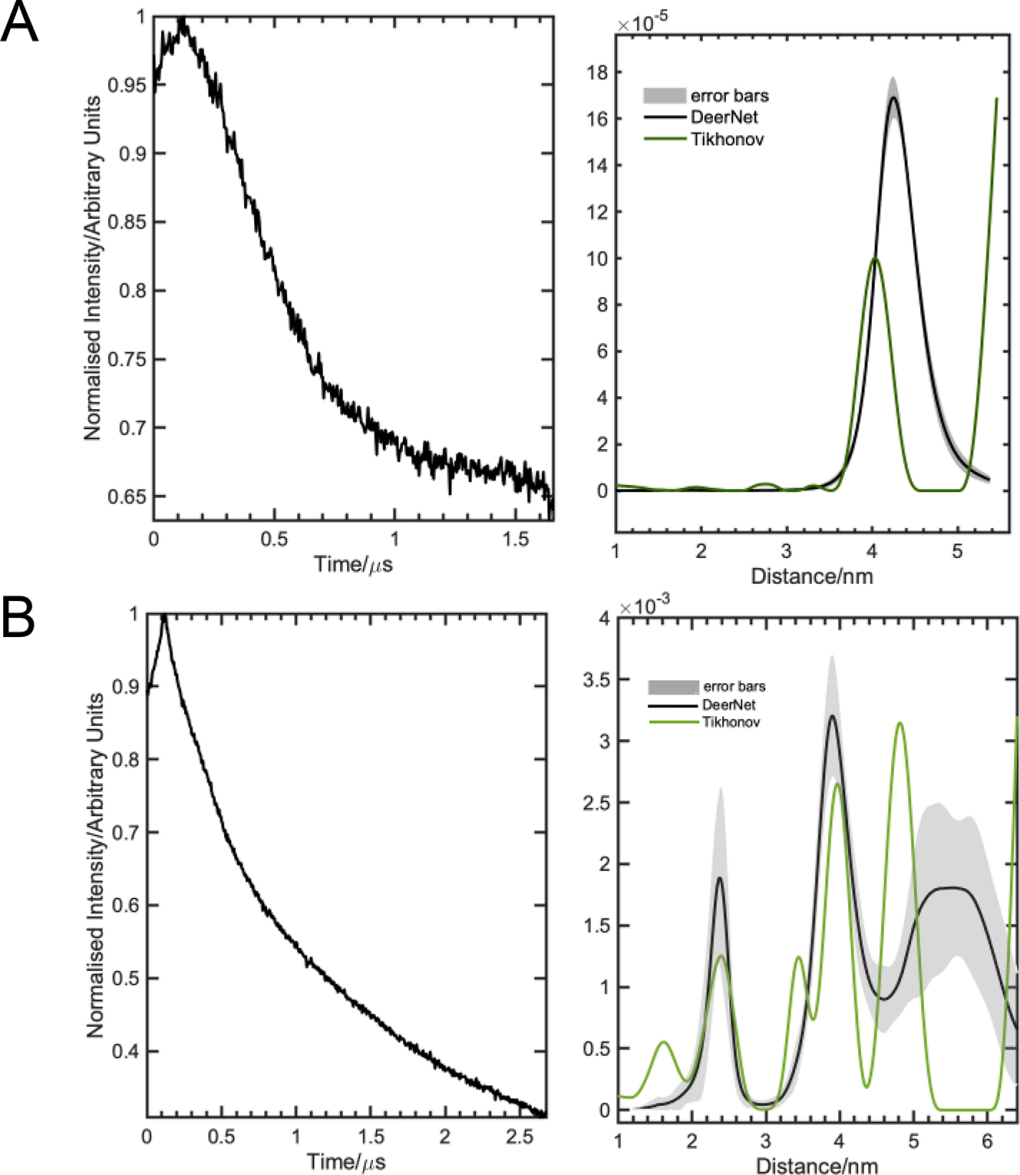
Background-subtracted X-band PELDOR time traces (left-hand panels) and comparisons of distance distributions output by Tikhonov and DEERNet analysis methods (right-hand panels) for the three-domain (A) *Ralstonia pickettii* and (B) *Hyphomicrobium denitrificans* copper-containing nitrite reductases. The PELDOR distance distribution validation is presented here by using the gray error bars.

PELDOR data for the *Hd*NiR “core” when analyzed by using Tikhonov regularization show three inter-copper distances with the major distance at 42.5 Å (Figure 2D). Similar to the interpretation of the *Rp*NiR “core” PELDOR spectroscopic data and as previously stated by van Wonderen et al.,^3^ we hypothesize that this distance is an accumulation of the T1–T1Cu and the longer T1–T2Cu distance. The minor distances recorded at 20.0 and 27.0 Å, which represent the T2–T2Cu and the shorter T1–T2Cu distances, respectively, are no longer present when PELDOR data were analyzed by the DEERNet method and could be the result of background fitting errors or artifacts. It is noteworthy to mention that the intensity of these peaks observed at 20 and 27.3 Å when the Tikhonov regularization method of analysis is used (Figure 2D) is significantly lower than that observed for the major peak at 42.5 Å, which is plausibly due to the low incorporation of the T2Cu (Figures S11 and S12).

Currently, there is no X-ray structure nor is there any mechanistic understanding of the *Hd*NiR “core” (however, classical CuNiR steady-state activity assays show this deconstructed protein is active). Therefore, we cannot make a comparison between the solution and the crystal structures of this protein. However, we note that the inter-copper distances from our *Hd*NiR “core” analysis are far shorter than the distances seen in the crystallographic structure of the full-length *Hd*NiR protein (the T2–T2Cu and the shorter T1–T2Cu distances are ∼9 Å shorter in the PELDOR data when compared to the full-length *Hd*NiR crystal structure; Figure 1B), suggesting a closer proximity between the monomeric units of the *Hd*NiR “core” trimer. As a number of critical CuNiR structural elements (i.e., one of the T2Cu coordinating His residues, the substrate access channel, and the putative proton channel) are dictated by the trimeric state of the protein,^21,25,26^ our PELDOR data of both three-domain CuNiR “core” portions complement crystallographic studies.^25^ These findings likely have implications for mechanistic understandings of the proton delivery pathway, the substrate access channel, and the electron transfer pathways in these CuNiRs.

Next, X-band PELDOR experiments were performed on the three-domain *Rp*NiR and the *Hd*NiR proteins to map inter-copper distributions. The continuous-wave (Figures S3 and S4), two-pulse, field-swept echo-detected EPR spectra (Figures S7 and S8), raw PELDOR data, and validation of distance distribution (Figures S13–S21) of the *Rp*NiR and the *Hd*NiR proteins are provided in the Supporting Information. Low T2Cu incorporation (∼30%) was seen in these full-length proteins—a result which could not be altered by addition of surplus copper and influenced the PELDOR signals associated with the T2Cu (Figure 3).

In contrast to the *Rp*NiR “core”, only three inter-copper distances could be observed in our PELDOR data recorded on the full-length *Rp*NiR protein (six distances seen in the *Rp*NiR “core”; Figure 2C). The major distance recorded at 40.0 Å is likely to arise from both the inter-T1Cu and the longer T1–T2Cu distances,^3^ while the distances recorded at 33.9 and 26.9 Å are interpreted here as the short T1–T2Cu distance and the T2–T2Cu distance, respectively. It should be noted that the distances at 33.9 and 26.9 Å are no longer present when the time axis is stretched up to 2.1–2.2 μs (Figures S15–S18), which possibly indicates these inter-copper distances are due to error in the background correction. This is further supported by the neural network (DEERNet) analysis (in which the errors due to user-defined parameters are no longer observed), where these distances are also absent.^31,32^ An additional distance at 19.4 Å was observed in PELDOR measurements performed on the *Rp*NiR protein when analyzed by using the Tikohnov regularization method. This may be the intramonomer T1–T2Cu distance, but because of the nature of the PELDOR technique, this distance is too short to be determined accurately.^4^ As the PELDOR measurements were collected at the *g* perpendicular region, and data show a similar modulation depth to that observed in the *Rp*NiR “core” (Figure 2), we could rule out possible orientation and multispin effects in our analysis.^36,37^ We infer that the change from six inter-copper distances in the isolated “core” portion of *Rp*NiR (symbolic of two predominant conformations; Figure 2C) to minimal inter-copper distances in the full-length *Rp*NiR (signifying a single predominant conformation; Figures 3A and S13–S18) represents a change in the conformational landscape of the “core” region of *Rp*NiR due to cytochrome *c* tethering. We have recently shown how tethering influences catalysis in *Rp*NiR through a number of unforeseen mechanisms,^24^ and the PELDOR data presented here provide insights into how the heme domain fusion influences the solution structure of the protein by causing the “core” region to occupy a single, but catalytically “beneficial” conformational state. On the basis of these data and the X-ray structure,^25^ we suggest that the change in the “core” conformational landscape in *Rp*NiR is due to the tether linking the heme domain to the “core” region, which wraps from one monomeric unit around the “core” region of the adjacent monomer (Figure 1A). The PELDOR data performed on the full-length *Rp*NiR protein also reveal significant changes to the distance distribution pattern compared to that of the *Rp*NiR “core”, and this plausibly implies that the predominant trimeric form of *Rp*NiR state in solution is different from that shown in the X-ray structure—a result which has implications for mechanistic understanding of the cytochrome *c*-tethered CuNiR proteins. We note that the absence of longer distances in the PELDOR data, arising from the ferric heme–Cu^II^ centers in the *Rp*NiR protein, is due to the excitation/resonator bandwidth, which is smaller than the separation between the heme parallel (2100 G) and cupric EPR signals (3330 G) (see Figure S19). Our PELDOR analysis (Figures 3A and S13–S18) for the full-length *Rp*NiR protein predominantly displayed a distance distribution at ∼41.2 Å. PELDOR traces were also collected at 3364 G by keeping the pump frequency at ∼9.673 GHz and detecting at −100 MHz (Figure S15)/+100 MHz (Figure S17) from this field (3364 G)/frequency (9.673 GHz). These two PELDOR traces were collected over the time axis of ∼2.2 μs, but the signal-to-noise ratio varies significantly, possibly due to the difference in the detecting frequency. In all three PELDOR traces of the *Rp*-NiR-WT, the copper–copper distance peak at ∼41.2 Å is predominantly observed when analyzing the full length PELDOR data. Moreover, it is highly dissimilar to the PELDOR data collected for the *Rp*NiR “core”, which show a broad distance distribution between 2 and 5 nm with defined distances at 30, 35, and 42 Å alongside a number of weak intensity peaks between 2 and 3 nm (Figures 2C, S9, S10, and S24).

In an effort to remove the complexity and/or deconvolute the various copper–copper distances in the “core” and full-length *Rp*-NiR proteins, the redox potentials of the T1Cu and T2Cu centers were exploited to selectively reduce a primary redox center of the CuNiRs—an approach similar to that of iDEER experiments. In addition, site-selective mutation and copper(II) supplement were controlled to remove the T2Cu center but encountered challenges to selectively remove one of the copper centers (Figure S25). The cw-EPR spectra of the “one-electron” reduced *Rp-*NiR-core sample show contributions from the residual “T1Cu” center and the T2-depleted sample showed incomplete removal of T2Cu (Figure S25). We have collected DEER data (Figure S26) on the “one-electron” reduced *Rp*NiR sample; however, the signal-to-noise ratio was poor. The analysis using Tikhonov regularization and the extracted distance distribution are included in the Supporting Information (Figure S26). The selective one-electron reduction of the T1Cu center has removed the predominant distance distribution peak at 4.12 nm and shows a distance at ∼3.5 nm, consistent with one of the T1Cu–T2Cu distances present in the *Rp*NiR “core”. We could not perform similar DEER measurements on the full length *Rp*NiR protein as the potentials of the three redox centers are the same in this system.^30^

As data show that the *Rp*NiR “core” and full-length proteins have similar *T*_1_ and *T*_2_ spin relaxation times (Figure S23), the effects of relaxation on the differences in the observed distance distribution can be ruled out. As expected, the cw-EPR spectra (Figure S3, top) shows the presence of the C-terminal “partner-protein” heme. It can therefore be inferred that the observed differences in the distance distribution pattern for full length *Rp*NiR are likely to arise from the interactions of the heme center with the copper centers. As the heme is only 16.7 Å from the T1Cu, the spin coupling between these two centers could not be eliminated. Other possibilities such as different dynamical properties of the full-length protein compared to that of *Rp*NiR “core” cannot be excluded.

In contrast to *Rp*NiR proteins, the modulation depth for the *Hd*NiR full length protein is 4 times that observed in the *Hd*NiR “core”. This is due to increased number of spins (9 vs 18) contributing at this magnetic field.^36,37^ As expected, a vast number of inter-copper distances were detected when performing PELDOR spectroscopy on the full-length *Hd*NiR protein (Figure 3B). However, by analyzing the *Hd*NiR “core” data (Figure 2D), we were able to deconvolute the distance measurements associated with the “core” region from the full-length protein. We infer that the shoulder of the large intensity peak at 24.3 Å, centered on 20 Å, is likely to be associated with the T2–T2Cu distance within the “core” region, while the peak at 41 Å will have contributions from the T1–T1Cu distance and the longer T1–T2Cu distance within the “core” region (among other distance measurements associated with tethering of the azurin domain). The PELDOR analysis of *Hd*-NiR full length protein by the neural network method (DEERNet) shows additional broad distance distribution peaks between 5 and 6 nm, which were not observed in the Tikhonov analysis (green trace, Figure 3B). Interestingly, there are several long T2Cu(core)–T1Cu(azurin) and T1Cu(core)–T1Cu(azurin) inter-copper distances that are found in the *Hd*-NiR full length structure (Figure 1B): T2Cu(core)–T1Cu(azurin); 50.9, 58.3, and 60.8 Å; T1Cu(core)–T1Cu(azurin); 51.2, 58.1, and 58.9 Å—these broad distance distributions between 5 and 6 nm might plausibly represent the collections of all these long distance ranges present in the HdNiR full length protein. Altogether, in the full-length structure of *Hd*NiR, there are a multitude of distances seen in the crystal structure. Many of these distances are seen in our PELDOR data (Figures 3B, S20, and S21), and it is probable in some cases that signals may have accumulated to form the same peak in our distance distribution analysis. This makes it challenging to assign specific distances seen in the PELDOR data to individual inter-copper distances and thus build a more complete solution-state structure. It is evident from the comparisons of distance distribution outputs of the “core” and full length *Rp*NiR and *Hd*NiR protein samples (Figure S24) that the conformational changes of the *Rp*NiR-WT adapt a more compact geometry, whereas the *Hd*NiR-WT PELDOR results show additional long copper–copper distances consistent with the formation of the hexameric structure for the *Hd*NiR-WT protein.

In conclusion, using a combination of protein reverse engineering and PELDOR spectroscopy, we have been able to obtain the solution state inter-copper distribution for two three-domain partner protein-linked copper-containing nitrite reductases and the corresponding deconstructed two-domain “core” regions of these proteins. Much of the mechanistic understanding of copper-containing nitrite reductases, including details of the substrate entry channel, the proton delivery pathways, the electron transfer pathway and the coordination of the active site copper ions, has been obtained by using crystallographic data.^21,25,26^ Our data presented here complement these data and show that in solution three-domain CuNiRs exist in a range of conformational states. These data, which also point out the beneficial role of tethering in three-domain NiR catalysis, have profound implications for mechanistic understanding of the copper nitrite reductases and support mounting evidence^27^ for a role for protein dynamics in the catalytic cycle of this enzyme family and other multicenter metalloenzymes.

## Supporting Information Available

Experimental procedures along with experimental and simulated electron paramagnetic resonance spectroscopic data (PDF)

## Notes

Additional research data (PELDOR and cw-EPR raw data) supporting this manuscript are available from “FigShare” and can be viewed/downloaded at 10.48420/c.6094824.v1.
