# Supplementary material for: Solution-State Inter-Copper Distribution of Redox Partner-Linked Copper Nitrite Reductases: A Pulsed Electron–Electron Double Resonance Spectroscopy Study
Source: J Phys Chem Lett. 2022 Jul 22;13(30):6927–34. doi: 10.1021/acs.jpclett.2c01584 (PMC9358711; doi:10.1021/acs.jpclett.2c01584)
Supplement: Supplementary file 1 — jz2c01584_si_001.pdf [file jz2c01584_si_001.pdf]

Supplementary Information:

Solution-State Inter-Copper Distribution of Redox Partner-Linked Copper Nitrite Reductases: A Pulsed Electron-Electron Double Resonance Spectroscopy Study

Tobias M. Hedison[a][b], Andreea I. Iorgu[a], Donalto Calabrese[a], Derren J. Heyes[a], Muralidharan Shanmugam*[a], and Nigel S. Scrutton*[a][b]

[a] Manchester Institute of Biotechnology, School of Chemistry, University of Manchester, UK, M1 7DN.
[b] BBSRC and EPSRC funded Future Biomanufacturing Research Hub, Manchester Institute of Biotechnology and School of Chemistry, University of Manchester, UK, M1 7DN.
* corresponding authors: nigel.scrutton@manchester.ac.uk and muralidharan.shangmugam@manchester.ac.uk

Experimental Section

Materials

All reagents were of analytical grade and were purchased from Sigma-Aldrich, unless otherwise stated. To ensure all the copper ions bound to the nitrite reductase proteins were EPR active (i.e, in the Cu^{II} oxidation state), all protein samples were oxidised by addition of excess potassium ferricyanide (Fe^{III}/Fe^{II} of approx. 450 mV)[1] and subsequently, passed down a Bio-Rad Econo-Pac 10DG-desalting column equilibrated with the desired buffer.

Recombinant proteins

Ralstonia picketti copper nitrite reductase expression and purification

Recombinant C-terminal His-tagged *Ralstonia picketti* copper nitrite reductase (*Rp*NiR) was expressed in C41(DE3) *Escherichia coli* cells and purified to homogeneity using published protocols.[2] Briefly, pET22b plasmids containing the codon optimised gene encoding the *Rp*NiR protein were transformed into C41(DE3) *E.coli* cells and grown in 0.5 L cultures of Terrific Broth media. Once an OD_{600} of 0.6 was attained, protein synthesis was induced with the addition of 0.4 mM IPTG. To ensure high levels of heme *c* and copper incorporation in *Rp*NiR, all cultures were supplemented with 0.3 mM δ-Aminolevulinic acid and 0.1 mM $CuSO_4$. Cells were grow for >12 hours following induction. Cell pellets were harvested using centrifugation and were stored at -20 ^{o}C before purification.

*Rp*NiR was purified by nickel affinity chromatography. Cell pellets, dissolve in 40 mM MOPS (pH 7.8) buffer solutions supplemented with 150 mM NaCl were sonicated in the presence of DNase, 1 mM $MgCl_2$ and protease inhibitor cocktail tablets. Cell lysates were clarified by centrifugation and passed down a HisTrap HP histidine-tagged protein purification columns from GE Healthcare buffered with 40 mM MOPS (pH 7.8) and 150 mM NaCl. Nickel affinity columns were washed with the same buffer, supplemented with 40 mM imidazole. *Rp*NiR was eluted from the nickel affinity column using buffered solutions containing 40 mM MOPS (pH 7.8), 150 mM NaCl and 250 mM imidazole. Using a Bio-Rad Econo-Pac 10DG-desalting column, purified *Rp*NiR samples eluted from the nickel affinity columns were buffer exchanged into 50 mM Tris-HCl (pH 7.8), 200 mM NaCl and 1 mM DTT for subsequent purification steps. To remove C-terminal His-tags, Tobacco Etch Virus (TEV) protease was incubated with the *Rp*NiR protein >12 hours at room temperature. A nickel affinity column (GE Healthcare) was used to separate the cleaved His-tags from the *Rp*NiR proteins. The purified *Rp*NiR protein was supplemented with copper ions by incubating it in 50 mM Tris-HCl (pH 7.8), 200 mM NaCl and 0.1 mM $CuSO_4$ for approximately 2 hours. Before storage, *Rp*NiR was desalted into 50 mM Tris-HCl (pH 7.8) buffer supplemented with 200 mM NaCl and passed down a gel filtration column, which showed as previously demonstrated that the *Rp*NiR is trimeric.[3] *Rp*NiR was stored at -80 ^{o}C.

Hyphomicrobium denitrificans copper nitrite reductase expression and purification

Recombinant N-terminal His-tagged *Hyphomicrobium denitrificans* copper nitrite reductase (*Hd*NiR) lacking the N-terminal leader sequence was cloned into pETM-11 between the *NcoI* and the *XhoI* sites (Geneart, Thermofisher). pETM-11 plasmids containing the codon optimised genes for the *Hd*NiR proteins were transformed into BL21(DE3) *Escherichia coli* cells, which were grown in 0.5 L cultures of Terrific Broth media. Once an OD_{600} of 0.6 was reached, protein production was initiated by addition of 0.3 mM IPTG. To increase copper ion incorporation in *Hd*NiR, cell cultures were incubated with 0.1 mM $CuSO_4$. Cells were left to grow overnight for >12 hours and were harvested by centrifugation. Cell pellets were stored at -20 ^{o}C before subsequent purification steps.

*Hd*NiR was purified to homogeneity using nickel affinity chromatography by following the same protocols used for the *Rp*NiR protein. *Hd*NiR was shown to be to run in a hexameric state on a gel filtration column, as previously shown.[4] *Hd*NiR was stored at -80 ^{o}C.

Ralstonia picketti copper nitrite reductase 'core' expression and purification

The 'core' region of *Ralstonia picketti* copper nitrite reductase (*Rp*NiR 'core') was expressed and purified using published protocols.[2] Briefly, the pETM-11 plasmid containing the gene for the *Rp*NiR 'core' were transformed into BL21(DE3) *E. coli* cells and grown in 0.5 L of Terrific Broth media. Protein synthesis was induced when an OD_{600} of 0.6 was reached by the addition of 0.3 mM IPTG. 0.1 mM $CuSO_4$ was added to cultures after reaching this stationary phase to increase the copper loading of the *Rp*NiR 'core' protein. Cells were grown for >12 hours and harvested using centrifugation. Cell pellets were stored at -20 ^{o}C before purification.

The *Rp*NiR 'core' protein was purified by nickel affinity chromatography using similar methods mentioned above for the *Rp*NiR and *Hd*NiR proteins. Likewise, the N-terminal His-tags were removed from the protein using TEV protease. The *Rp*NiR 'core' protein was shown to run as a trimer on gel filtration column, as previously shown.[2] The *Rp*NiR 'core' was stored at -80 ^{o}C.

Ralstonia picketti copper nitrite reductase 'cytochrome c' expression and purification

The 'cytochrome *c*' region of *Ralstonia picketti* copper nitrite reductase (*Rp*NiR 'cyt *c*') was expressed and purified using published protocols.[2] Briefly, the pET22b plasmid containing the gene for the *Rp*NiR 'cytochrome *c*' were transformed into C41(DE3) *E. coli* cells and grown in 0.5 L of Terrific Broth media. Protein synthesis was induced when an OD_{600} of 0.6 was reached by the addition of 0.3 mM IPTG. 0.1 mM $CuSO_4$ and 0.3 mM δ-Aminolevulinic acid was added to cultures after reaching this stationary phase to increase the copper and heme *c* loading of the *Rp*NiR 'cytochrome *c*' protein. Cells were grown for >12 hours and harvested using centrifugation. Cell pellets were stored at -20 ^{o}C before purification.

The *Rp*NiR 'cytochrome *c*' proteins were purified by nickel affinity chromatography using similar methods mentioned above for the *Rp*NiR protein. A copper-loading step was omitted from this purification. Likewise, the N-terminal His-tags were removed from the protein using TEV protease. The *Rp*NiR 'cytochrome *c*' protein was shown to run as a monomer on gel filtration column, as previously shown.[2] The *Rp*NiR 'cytochrome *c*' was stored at -80 ^{o}C.

Hyphomicrobium denitrificans copper nitrite reductase 'core' expression and purification

The codon optimised recombinant 'core' region of *Hyphomicrobium denitrificans* copper nitrite reductase (*Hd*NiR 'core') was cloned in pETM-11 between the *NcoI* and the *XhoI* sites. N-terminal His-tagged *Hd*NiR 'core' was expressed and purified using published protocols for the *Rp*NiR 'core' protein. Briefly, the pETM-11 plasmid containing the gene for the *Hd*NiR 'core' were transformed into BL21(DE3) *E. coli* cells and grown in 0.5 L of Terrific Broth media. Protein synthesis was induced when an OD_{600} of 0.6 was reached by the addition of 0.3 mM IPTG. 0.1 mM $CuSO_4$ was added to cultures after reaching the stationary phase to increase the copper loading of the *Hd*NiR 'core' protein. Cells were grown for >12 hours and harvested using centrifugation. Cell pellets were stored at -20 ^{o}C before purification.

The *Hd*NiR 'core' proteins were purified by nickel affinity chromatography using similar methods mentioned above for the *Rp*NiR 'core' protein. Likewise, the N-terminal His-tags were removed from the protein using TEV protease. The *Hd*NiR 'core' protein was shown to run as a trimer on a gel filtration column. The *Hd*NiR 'core' was stored at -80 ^{o}C.

Tobacco etch virus (TEV) protease expression and purification

The N-terminal His-tag tobacco etch virus (TEV) protease was expressed and purified by following previously published protocols.[5]

Electron paramagnetic resonance (EPR)

Electron paramagnetic resonance (EPR) measurements were carried out using a Bruker ELEXSYS-500/580 X-band EPR spectrometer operating in both continuous-wave (CW) and pulsed modes, equipped with an Oxford variable-temperature unit and ESR900 cryostat with Super High-Q resonator. EPR measurements were performed on approximately 900 μM of monomeric NiR (300 μM of trimeric NiR/150 μM of hexameric NiR) dissolved in 50 mM potassium phosphate buffers supplemented with 10 % glycerol. To increase the pulsed electron-electron double resonance (PELDOR) measurement window (time-domain, *vide infra*), both the buffer and the glycerol used for these measurements were deuterated. EPR samples were placed in 4 mm quartz capillary tubes (Wilmad-LabGlass) and frozen in liquid nitrogen. Samples were stored in liquid nitrogen until the measurements were conducted on them.

The X-band EPR tubes were then transferred into the EPR probehead, which was pre-cooled to 20 K. The low-temperature cw-EPR spectra were measured at 20 K. A microwave power of 30 dB (0.2 mW) and modulation of 5 G appear to be optimal for recording the EPR spectra of full-length (3-domain) and 'core' regions of the *Rp*NiR and *Hd*NiR proteins. The low temperature EPR spectra were acquired using the following conditions: sweep time of 84 s, microwave power of 0.2 mW, time constant of 41 ms, average microwave frequency of 9.386 GHz and modulation amplitude of 5 G.

X-band pulsed-EPR and PELDOR measurements were performed at 10 K, using the pulsed-mode with a dielectric resonator (ER4118X-MD-5) and a closed cryostat system (Bruker). Echo-detected field-swept spectra were measured using the following pulse sequence: t_p−τ−2t_p−τ−echo with $t_p(\pi/2)$ = 16 ns, τ = 0.2 μs, pulse repetition rate of 5 ms. The PELDOR experiments were performed using the pulse sequences, as indicated in Scheme S1:

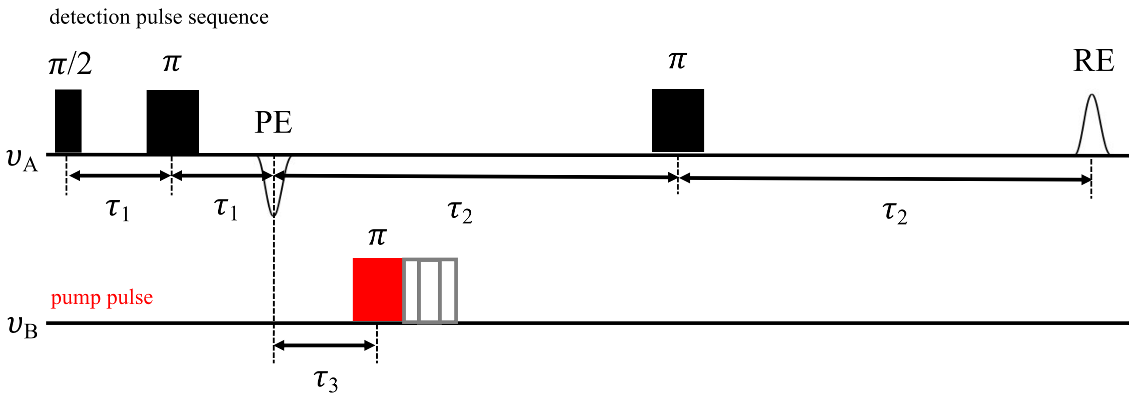

Scheme S1. Pulse sequences for PELDOR experiments carried at 10 K. The positions of the primary (PE) and refocused (RE) echoes are marked in the pulse sequences.

The π/2 and π pulses of the detection sequence had lengths of 16 and 32 ns, and the pump pulse was 14 ns long. The π/2 pulse was phase-cycled to eliminate the receiver offsets. The Δν (ν_{det} − ν_{pump}) was ± 100 MHz. The pulse separations, τ_1 and τ_3, were 200 and 100 ns, respectively, and the echo signal was integrated using a video amplifier bandwidth of 20 MHz. The τ_2 for the various constructs were 2.6 μs for the full-length *Hd*NiR and the *Hd*NiR 'core' region, 1.6 μs and 2.2 μs for the full-length *Rp*NiR, and 2.8 μs for the *Rp*NiR 'core'. The pump pulse was stepped out by 4 ns for a given τ_2 in time-domain axis. The PELDOR data were analysed using DeerAnalysis2022[6] and DEERNet Spinach SVN Rev 5662.[7,8] Analysis of the cw-EPR spectra was performed using the EasySpin toolbox (5.2.35) for the Matlab program package.[9] The raw-PELDOR data and validation of distance distribution for various constructs of *Rp*NiR and *Hd*NiR proteins are given below (**Figures S9-S21**). When conducting PELDOR analysis, background deconvolution is not exact and can introduce error. This error can often dominate the error in the resulting distance distributions. To overcome this, the background starting time (500-1500 ns), background dimensionalities (between 2-3) and the white noise (0.003-1.5) were varied systematically within a limit. The resultant 'n' form factors obtained were subjected to Tikhonov regularisation and the distance distributions obtained (lower/upper limit and mean value) are plotted in

Figures S9-S21. The PELDOR data displayed in the main text (*Hd*NiR full-length enzyme was analysed using DEERNet in the Spinach 2.5.5449 distribution)[7] were also analysed using DEERNet (neural network), where the errors due to the user-defined parameters are no longer present. It is also noteworthy to bring it to the attention of the readers that the PELDOR time traces for the “core” and “full-length” proteins (*Rp*NiR and *Hd*-NiR) were stretched as long as possible (Cu-Cu distances range from 3.0 - 6.1 nm for both *Rp*-NiR and *Hd*-NiR proteins) in an effort to minimise the error in the background deconvolutions, which often introduce error in PELDOR analysis.

Results

Design of the *Hyphomicrobium denitrificans* copper nitrite reductase 'core' protein.

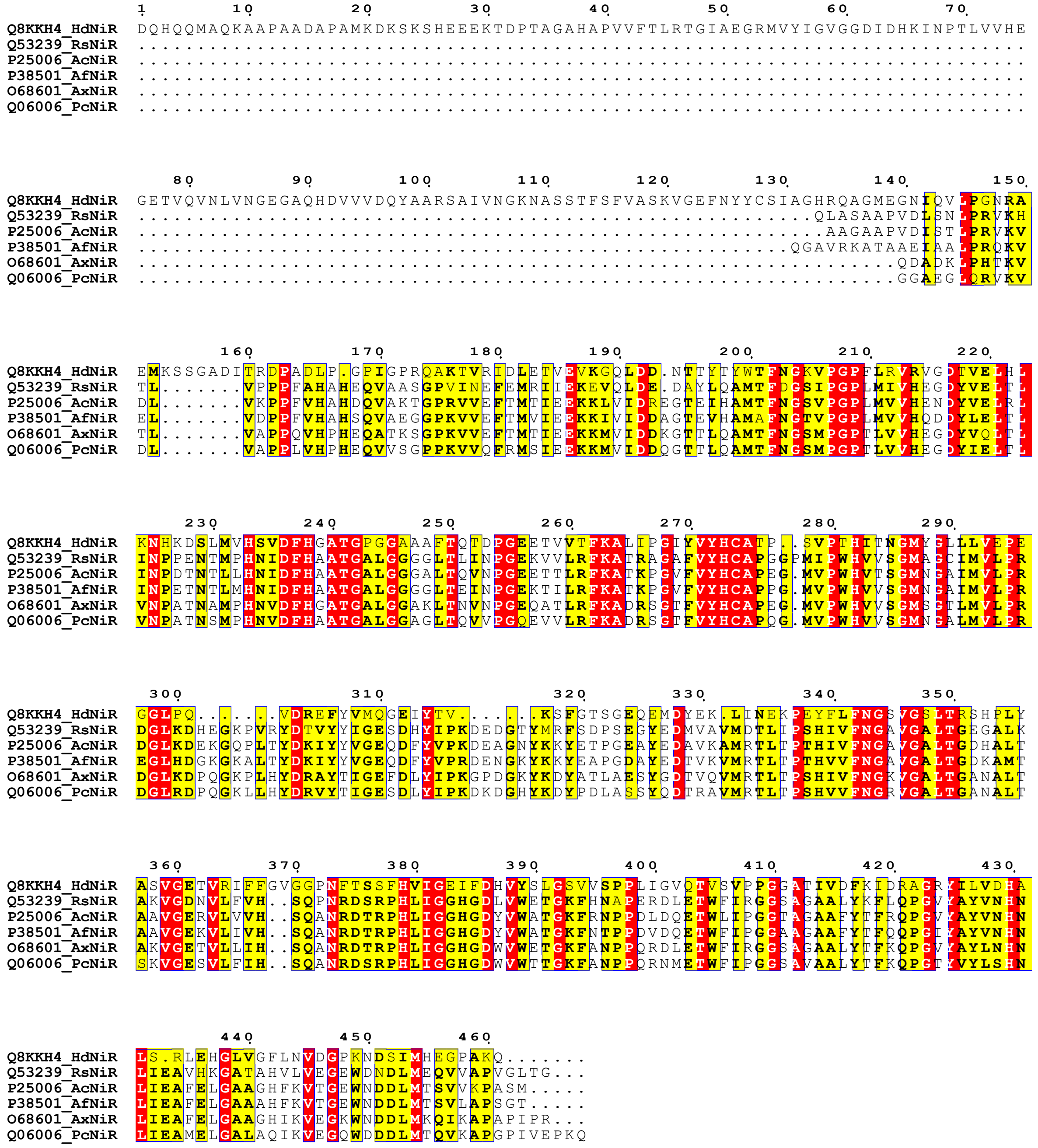

Figure S1. Sequence alignment of the *Hyphomicrobium denitrificans* copper nitrite reductase protein with a range of well-characterised 2-domain copper nitrite reductases. For simplicity, all the N-terminal periplasmic leader sequences have been omitted from the sequence alignment. The nitrite reductase proteins are shown with their abbreviated names along with their Uniprot accession codes. The residues that are conserved are shown in red, while those shown in yellow are partially conserved or share similar chemical properties. The alignment was performed with Clustals omega and the image was rendered with the ENDscript server.

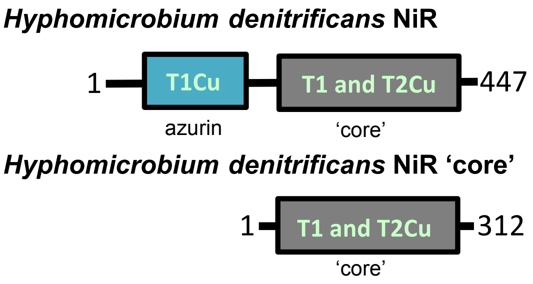

Figure S2. Schematic showing the full-length and the 'core' regions of the *Hyphomicrobium denitrificans* copper nitrite reductase. The 'core' region of *Hd*NiR is shown in grey and the N-terminal azurin domain is shown in blue.

Electron paramagnetic resonance (EPR) spectroscopy

PELDOR orientation selectivity

The dipolar coupling frequency of two interacting electron spins, S_A and S_B is described in Equation S1:

$$\omega_{ee} = \frac{\mu_0 g_A g_B \mu_B^2}{4\pi\hbar} \frac{1}{r_{AB}^3} (3cos^2\theta_{AB} - 1) \qquad \text{Eq. S1}$$

where μ_0 is the vacuum permeability, μ_B is the Bohr magneton, g_A and g_B are the g-values of the interacting electron spins (S_A and S_B), $\hbar$ is the reduced Planck constant, r_{AB} is the distance between the two spins, and θ_{AB} is the angle between the external magnetic field and the inter-spin vector (r_{AB}). The dipolar coupling frequency is dependent on both r_{AB} and θ_{AB}. In the case of flexible nitroxides, the relative orientations of the interacting spins are often randomized. Therefore, it is assumed that the microwave pulses in the PELDOR experiments uniformly excite most of the θ_{AB} at X-band. A well-known frequency distribution, called Pake pattern is produced from these experiments.[10] However, due to the large *g*-anisotropy of both type I (T1Cu) and type II (T2Cu) Cu^{II} centres present in *Rp*NiR and *Hd*NiR proteins, the Cu^{II}-Cu^{II} PELDOR experiments performed in this study fall under the category of 'orientation selection'. This leads to selective excitation of certain g-tensor components of the interacting Cu^{II} centres and a breakdown of the approximation of a Pake pattern as a frequency response.[10] Therefore, the Tikhonov method can no longer be applied in these investigations due to selective excitation of θ_{AB} by the applied microwave pulses.[6,11] In this case, it is often required to collect the PELDOR data at multiple magnetic fields across the EPR envelope to reliably extract the distance distribution between the interacting spins. As reported previously, to overcome this 'orientation selectivity', PELDOR data were collected in our investigation for the *Rp*NiR and *Hd*NiR constructs at frequencies close to $g_\perp$ (3310-3350 G), where contributions from molecules with a wide range of orientations are overlapped. This reduces the orientation selection at this magnetic fields and allows Tikhonov regularisation to be employed in this study.[12–14]

X-band continuous-wave EPR and Simulations of the RpNiR-WT and its 'core'

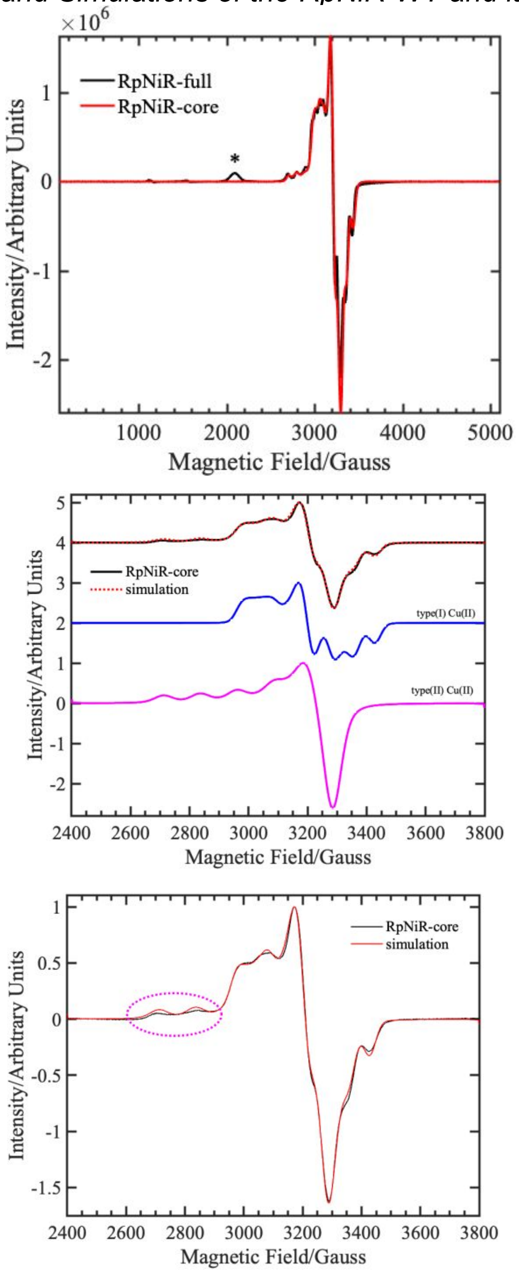

Figure S3. CW-EPR spectra of the full-length and 'core' regions of *Rp*NiR measured at 20 K (top) and its simulation (middle and bottom) as reported previously.[15] The EPR signal observed at 2100 G in the full-length *Rp*NiR, indicated by the black asterisk mark is due to the heme *c* cofactor, which is absent in the 'core' region of the *Rp*NiR protein. The other two broad EPR signals components of the heme *c* (**Figure S19**) are masked by the strong EPR signals arising from T1 and T2Cu centres. EPR spectrometer settings: microwave power = 30 dB; modulation amplitude = 5 G; time constant = 41 ms; conversion time = 41 ms; sweep time = 84 s: receiver gain = 60 dB; average microwave frequency = 9.386 GHz; temperature = 20 K. It is noteworthy to mention that the T2Cu contribution to the overall spectrum is ~ 50 % less compared to that of the T1Cu centre as indicated by the magenta dotted ellipse in the bottom trace. The relative contribution of the type(II) Cu(II) centre is even less in the 2-pulsed, field-swept EPR spectra (*vide infra*), approx. 30 %.

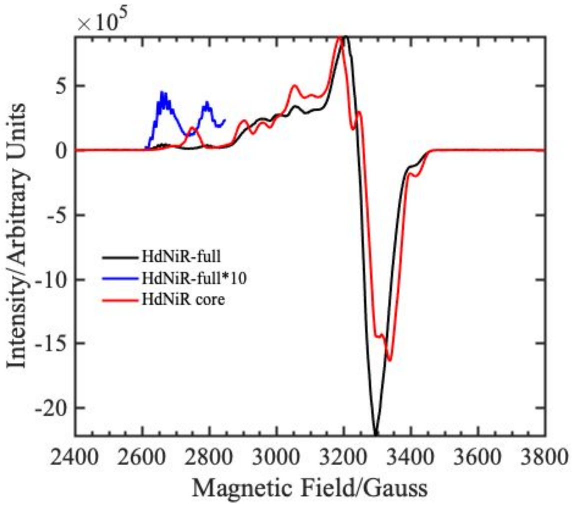

Figure S4. CW-EPR spectra of full length and the 'core' region of *Hd*NiR protein measured at 20 K. For clarity, the weak parallel features and super-hyperfine splittings arising from ^{63,65}Cu [$A_{||}$(^{63,65}Cu)] and ^{14}N nuclei in the full-length *Hd*NiR are expanded at the low field region between 2500-2900 G (blue trace). The observed super-hyperfine splittings (blue trace) are due to the coordinated ^{14}N nucleus of the histidine ligands to the Cu^{II} ion. *Conditions*: as in **Figure S3**.

Simulations of continuous-wave EPR spectra of the full-length HdNiR and *Hd*NiR *'core' enzymes*

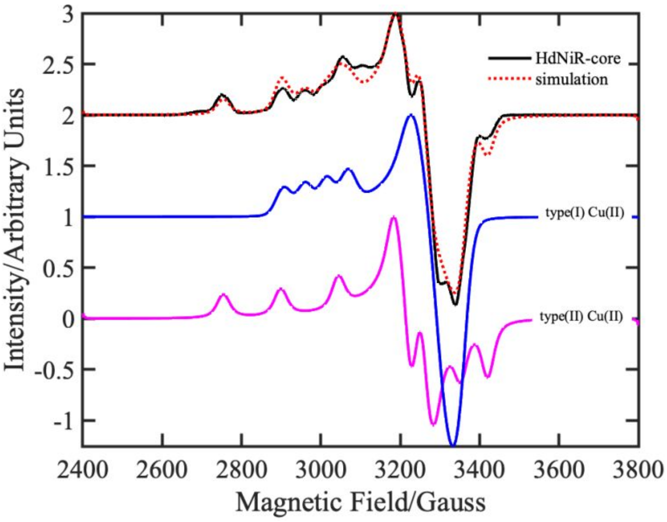

Figure S5. Experimental (black line) and simulated (red dotted line) EPR spectra of the *Hyphomicrobium denitrificans* copper containing nitrite reductase 'core' (*Hd*NiR 'core'). The EPR spectra were successfully simulated by considering two contributing, *S* = ½ spin species with the following spin-Hamiltonian parameters: T1Cu; *g* = [2.010 2.054 2.244], ^{63,65}Cu(A) = [35 50 170] MHz; line widths = [3.38 0.4] mT; weight = 0.5; T2Cu; *g* = [2.015 2.087 2.255], ^{63,65}Cu(A) = [180 10 458] MHz; line widths = [1.56 1.52] mT; weight = 0.5, which implies that both T1 and T2Cu centres have an equivalent occupancy in the *Hd*NiR core.

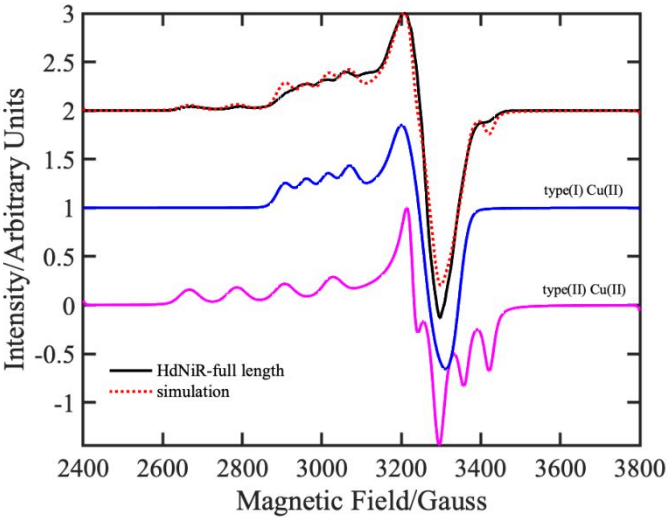

Figure S6. Experimental (black line) and simulated (red dotted line) EPR spectra of the *Hyphomicrobium denitrificans* copper containing nitrite reductase (*Hd*NiR full length). The EPR spectra were successfully simulated by considering two contributing, *S* = ½ spin species with the following spin-Hamiltonian parameters: T1Cu; *g* = [2.022 2.072 2.244], ^{63,65}Cu(A) = [40 50 170] MHz; line widths = [3.38 0.3] mT; weight = 0.4; T2Cu; *g* = [2.012 2.059 2.355], ^{63,65}Cu(A) = [170 15 395] MHz; 3 equally contributing, ^{14}N(A) = [5 5 40] MHz; line widths = [1.2 1.12] mT; weight = 0.22. For the *Hd*NiR full-length protein, the signal for the T2Cu (0.22 weight) is ~50 % of the T1Cu signal (0.4 weight).

2-pulse echo-detected field-swept EPR

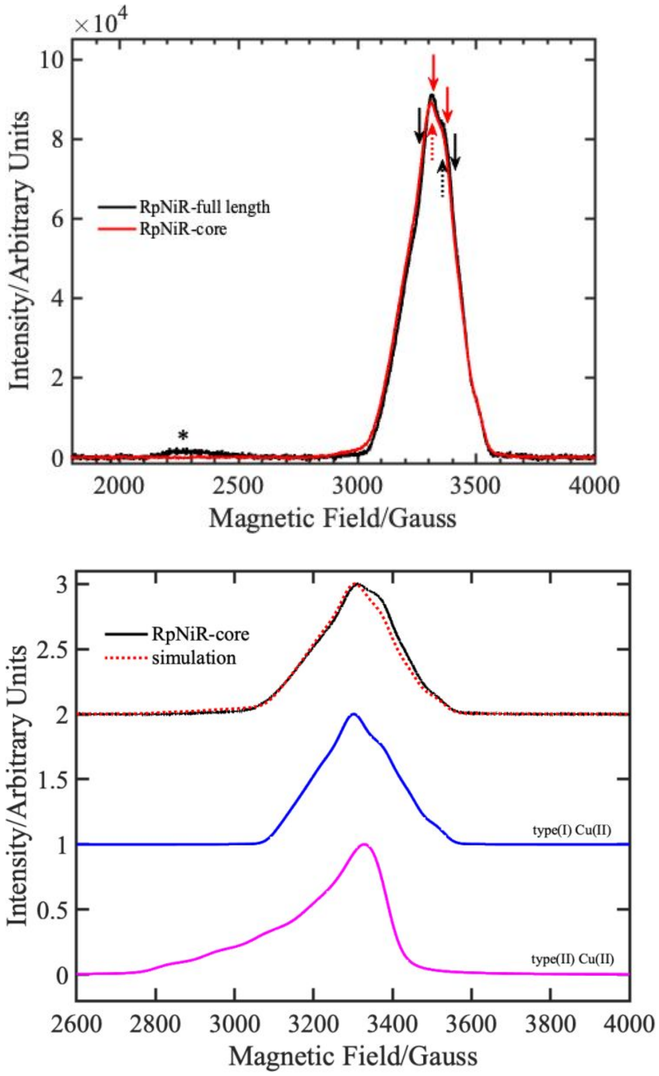

Figure S7. 2-pulse, echo detected, field-swept EPR spectra of the full-length and the 'core' regions of *Rp*NiR measured at 10 K (**top**). The positions of the probe (black arrows) and pump (red arrows) microwave pulses in the PELDOR experiments are indicated by the black and red arrows (dotted lines; *Rp*NiR core), respectively. The EPR signal observed at 2250 G, indicated by the black asterisk mark is due to the ferric heme *c* species, which is absent in the 'core' region of *Rp*NiR, consistent with the cw-EPR results. The PELDOR data collected at different pumping and detecting fields for the *Rp*NiR full length sample did not produce additional distances corresponding to longer ferric heme-Cu^{II} distances. EPR spectrometer conditions: π-pulse length = 32 ns, τ = 0. 2 μs, short repetition rate = 5 ms, average microwave frequency = 9.674 GHz. (**bottom**) Simulation of the *Rp*NiR 'core' using the spin-Hamiltonian parameters as reported previously.[15] The relative weight of the T2Cu(**II**) to that of the T1Cu(**II**) centre is ~ 26%. The discrepancy in weight between cw- and pulsed-EPR method is currently not known and is likely associated with the relaxation effects of the T1Cu and T2Cu centres.

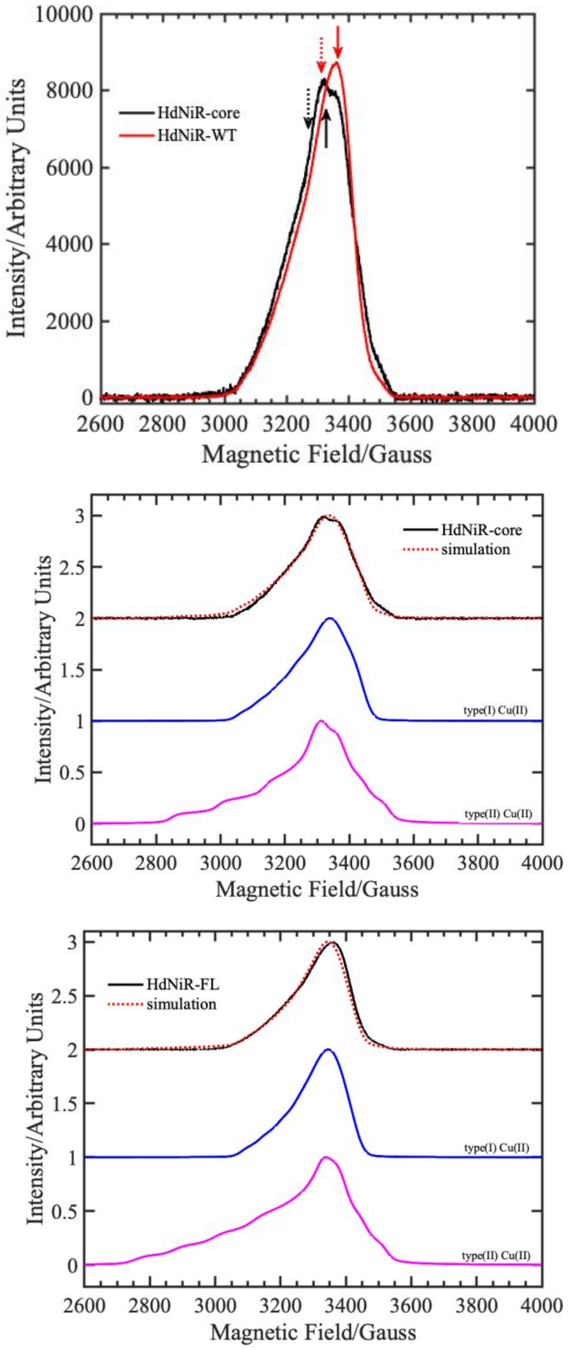

Figure S8. 2-pulse, echo detected, field-swept EPR spectra of the full-length and the 'core' region of *Hd*NiR measured at 10 K (**top**) and their simulations (**middle and bottom** traces). The positions of the probe and pump microwave pulses in the PELDOR experiments are indicated by the black and red arrows (dotted lines; *Hd*NiR core), respectively. EPR spectrometer conditions: π-pulse length = 32 ns, τ = 0. 2 μs, short repetition rate = 5 ms, average microwave frequency = 9.674 GHz. Simulations of the *Hd*NiR 'core' (**middle**) and *Hd*NiR full-length (**bottom**) using the spin-Hamiltonian parameters provided in Figures S5 and S6. From the modelling of the pulsed EPR spectra, it is clear that the relative weight of the T2Cu(II) to that of the T1Cu(II) centre is ~ 25-30%.

Four-pulse PELDOR spectrum and distance distribution of the RpNiR 'core'

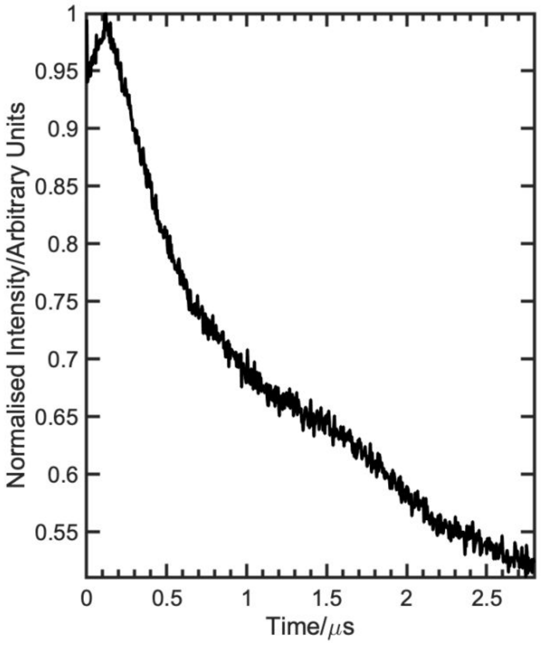

Figure S9. Four-pulse PELDOR spectrum of *Rp*NiR 'core'. The pulse separations and the lengths of the 'detection/pump' microwave pulses were as described in scheme **S1**. The spectrum was recorded at 3310 G with $\upsilon_{detection}$ = 9.5779 GHz and υ_{pump} = 9.6778 GHz; The Δv ($v_{det} - v_{pump}$) was --100 MHz, whereas in all other constructs, the Δv was $\pm$ 100 MHz. The DEER trace has been averaged over '8780' scans with the shot repetition time of 3060 μs. The validation of a distance distribution is given in **Figure S10** (linked to main text 2C)

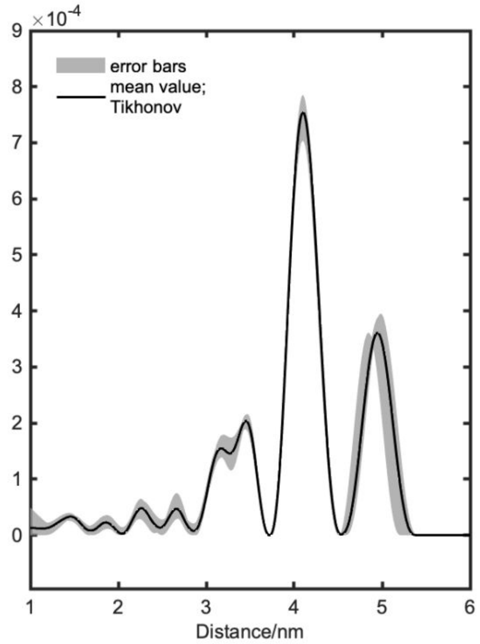

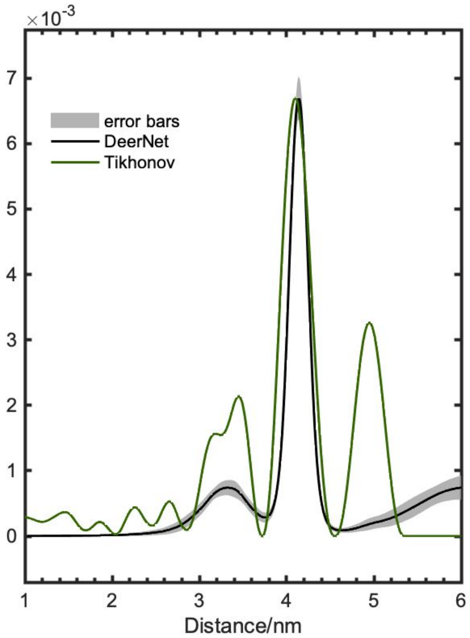

Figure S10. Validation of a distance distribution by Tikhonov regularisation method in the *Rp*NiR 'core' (**left hand panel**) and comparison of the distance distribution of the *Rp*NiR 'core' obtained by neural network (black trace; **right hand panel**) and Tikhonov regularisation methods (green trace; **right hand panel -** linked to main Figure 2C).

Four-pulse PELDOR spectrum and distance distribution of the HdNiR 'core'

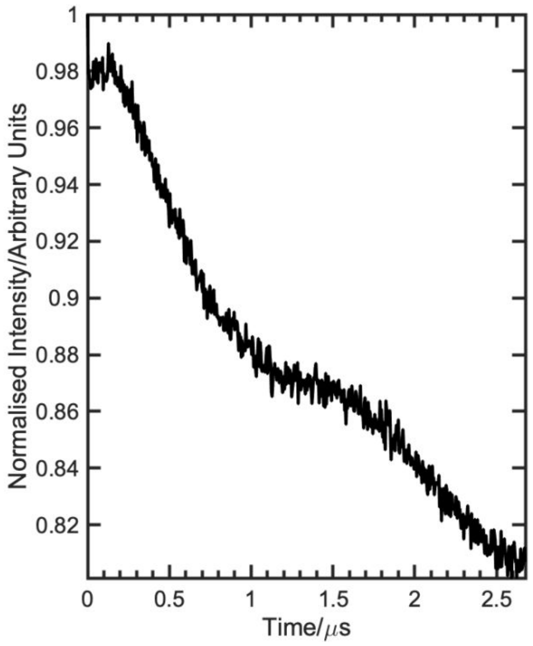

Figure S11. Four-pulse PELDOR spectrum of *Hd*NiR 'core'. The pulse separations and the lengths of the 'detection/pump' microwave pulses were as described in scheme **S1**. The spectrum was recorded at 3310 G with $\upsilon_{detection}$ = 9.7742 GHz and υ_{pump} = 9.6739 GHz; the Δv ($v_{det} - v_{pump}$) was +100 MHz. The DEER trace has been averaged over '21681' scans with the shot repetition time of 5000 μs. The validation of a distance distribution is given in **Figure S12** (linked to main Figure 2D).

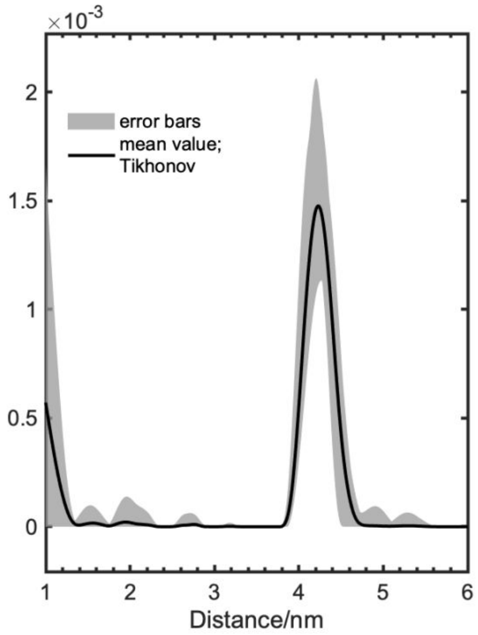

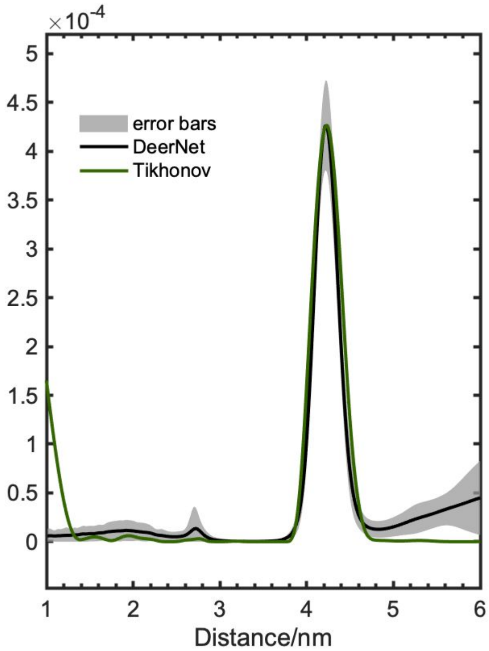

Figure S12. Validation of a distance distribution by Tikhonov regularisation method in *Hd*NiR 'core' (**left hand panel**) and comparison of the distance distribution of the *Hd*NiR 'core' obtained by neural network (black trace; **right hand panel**) and Tikhonov regularisation methods (green trace; **right hand panel -** linked to main Figure 2D;).

Four-pulse PELDOR spectrum and distance distribution of the full-length RpNiR

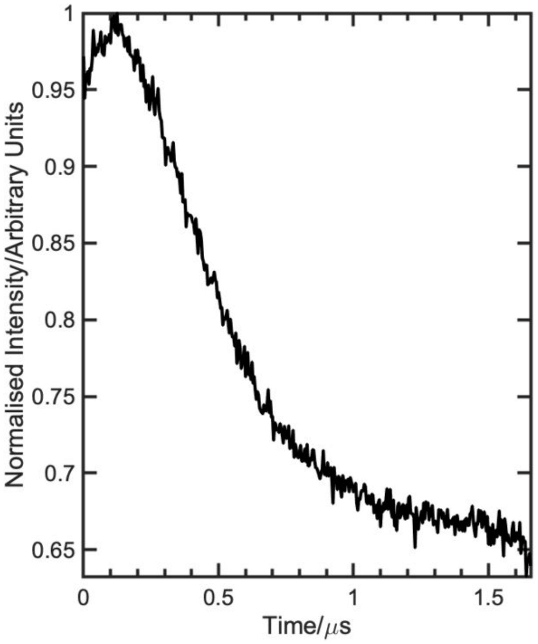

Figure S13. Four-pulse PELDOR spectrum of *Rp*NiR. The pulse separations and the lengths of the 'detection/pump' microwave pulses were as described in **Scheme S1**. The spectrum was recorded at 3310 G with $\upsilon_{detection}$ = 9.7728 GHz and υ_{pump} = 9.6728 GHz; the Δv ($v_{det} - v_{pump}$) was +100 MHz and the validation of a distance distribution is given in **Figure S14** (linked to main Figure 3A). The DEER trace has been averaged over '11930' scans with the shot repetition time of 3060 μs.

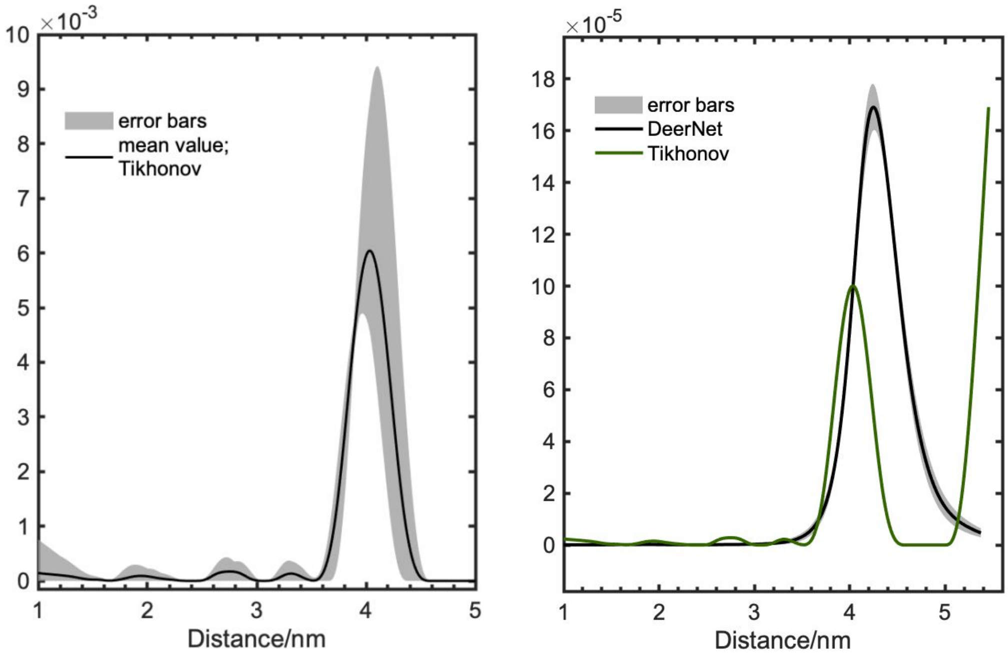

Figure S14. Validation of a distance distribution by Tikhonov regularisation method in *Rp*NiR-WT (**left hand panel**) and comparison of the distance distribution of the *Rp*NiR full-length obtained by neural network (black trace; **right hand panel**) and Tikhonov regularisation methods (green trace; **right hand panel-** linked to main Figure 3A;).

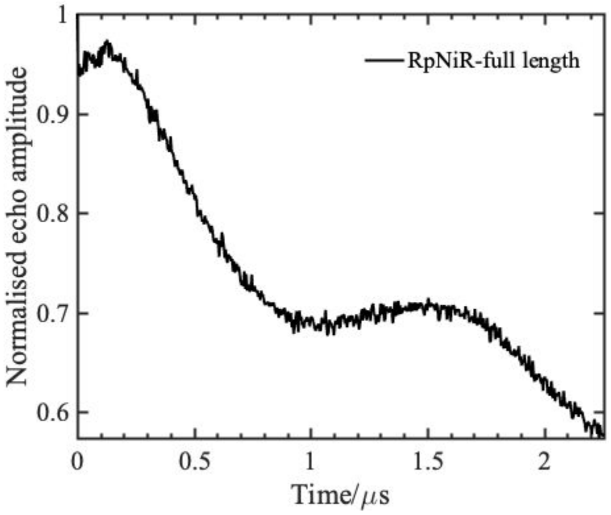

Figure S15. Four-pulse PELDOR spectrum of WT-*Rp*NiR. The pulse separations and the lengths of the 'detection/pump' microwave pulses were as described in scheme **S1**. The spectrum was recorded at 3364 G with $\upsilon_{detection}$ = 9.5757 GHz and υ_{pump} = 9.6757 GHz; the Δv ($v_{det} - v_{pump}$) was -100 MHz and the analysis performed using DeerAnalysis2015 is given in **Figure S16**. The PELDOR trace has been averaged over '11626' scans with the shot repetition time of 3060 μs.

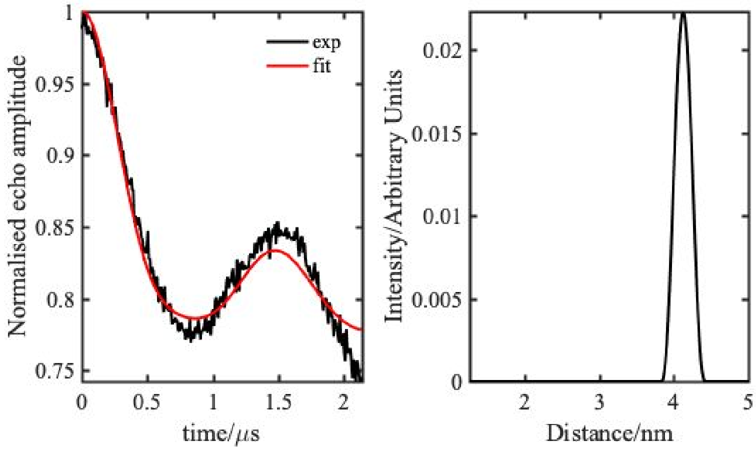

Figure S16. PELDOR time trace following background subtraction (left) and the corresponding distance distribution outputs (right) for the 3-domain copper-containing nitrite reductase, full-length *Rp*NiR.

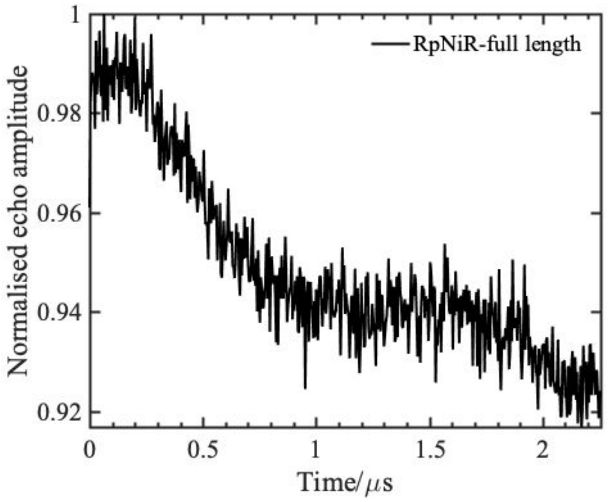

Figure S17. Four-pulse PELDOR spectrum of *Rp*NiR. The pulse separations and the lengths of the 'detection/pump' microwave pulses were as described in scheme **S1**. The spectrum was recorded at 3364 G with $\upsilon_{detection}$ = 9.7730 GHz and υ_{pump} = 9.6728 GHz; The Δv ($v_{det} - v_{pump}$) was -100 MHz and the distance distribution analysis performed using DeerAnalysis2015 is given in **Figure S18**. The PELDOR trace has been averaged over '23000' scans with the shot repetition time of 3000 μs.

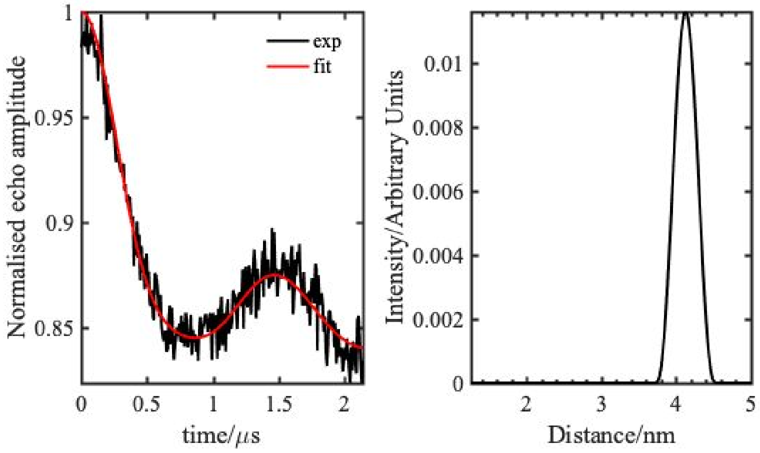

Figure S18. PELDOR time trace following background subtraction (left) and the corresponding distance distribution outputs (right) for the 3-domain copper-containing nitrite reductase, full-length *Rp*NiR.

X-band continuous-wave EPR of the RpNiR cyt c protein

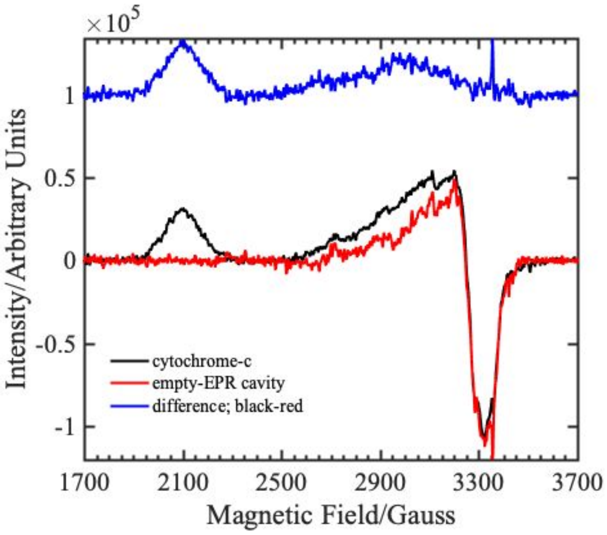

Figure S19. cw-EPR spectra of the *Rp*NiR cytochrome *c* protein measured at 20 K. *Conditions*: as in **Figure S3**.

Four-pulse PELDOR spectrum and distance distribution of the full-length HdNiR

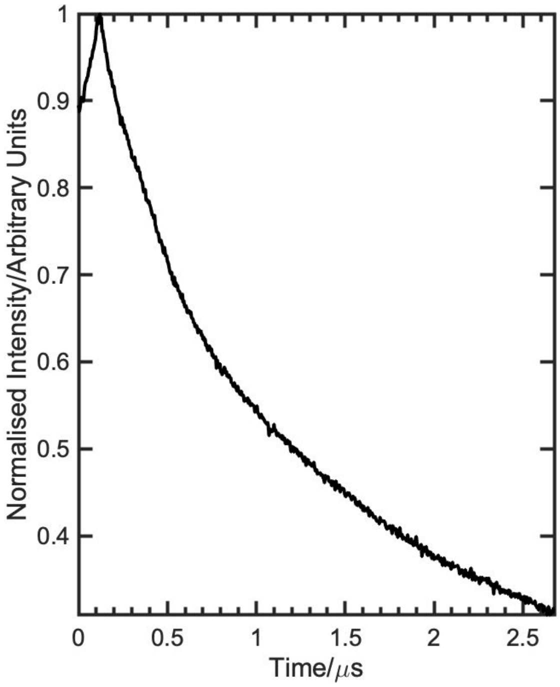

Figure S20. Four-pulse PELDOR spectrum of *Hd*NiR-WT. The pulse separations and the lengths of the 'detection/pump' microwave pulses were as described in scheme **S1**. The spectrum was recorded at 3335 G with $\upsilon_{detection}$ = 9.7752 GHz and υ_{pump} = 9.6752 GHz; the Δv ($v_{det} - v_{pump}$) was +100 MHz. The DEER trace has been averaged over '15100' scans with the shot repetition time of 5000 μs. The validation of a distance distribution is given in **Figure S21** (linked to main Figure 3B).

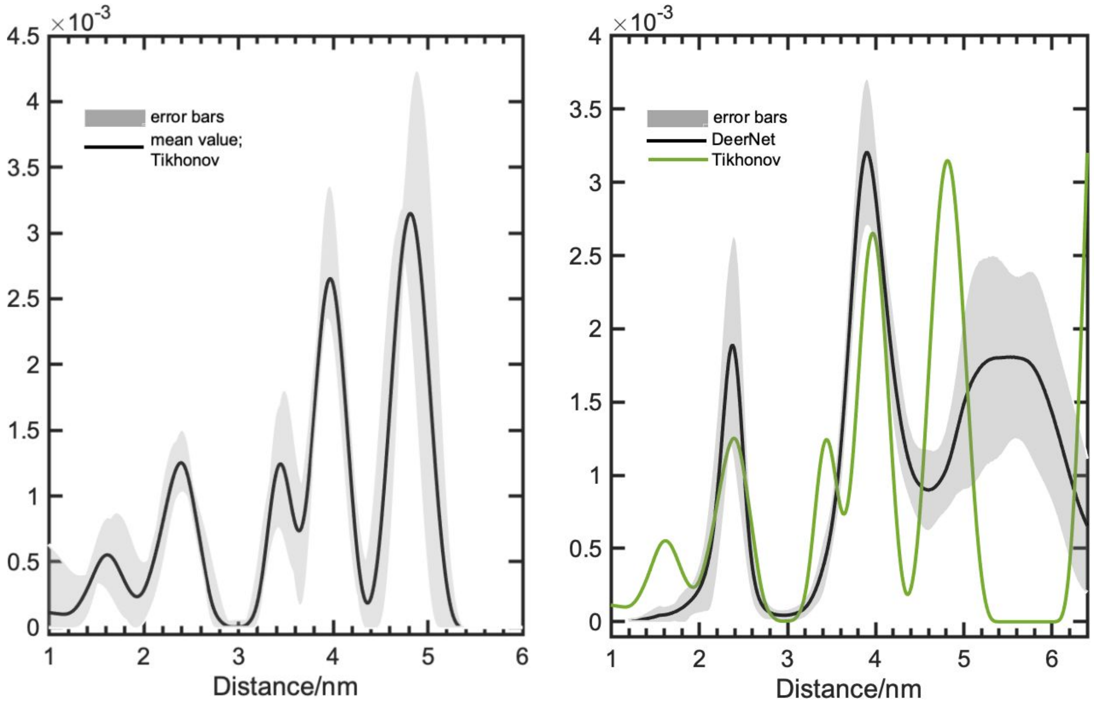

Figure S21. Validation of a distance distribution by Tikhonov regularisation method in *Hd*NiR-WT (**left hand panel**) and comparison of the distance distribution of the *Hd*NiR full-length obtained by neural network (black trace; **right hand panel**) and Tikhonov regularisation methods (green trace; **right hand panel-** linked to main Figure 3B;).

Relaxation times – T1-inversion recovery and phase-memory times of the 'core' and full-length (WT) proteins

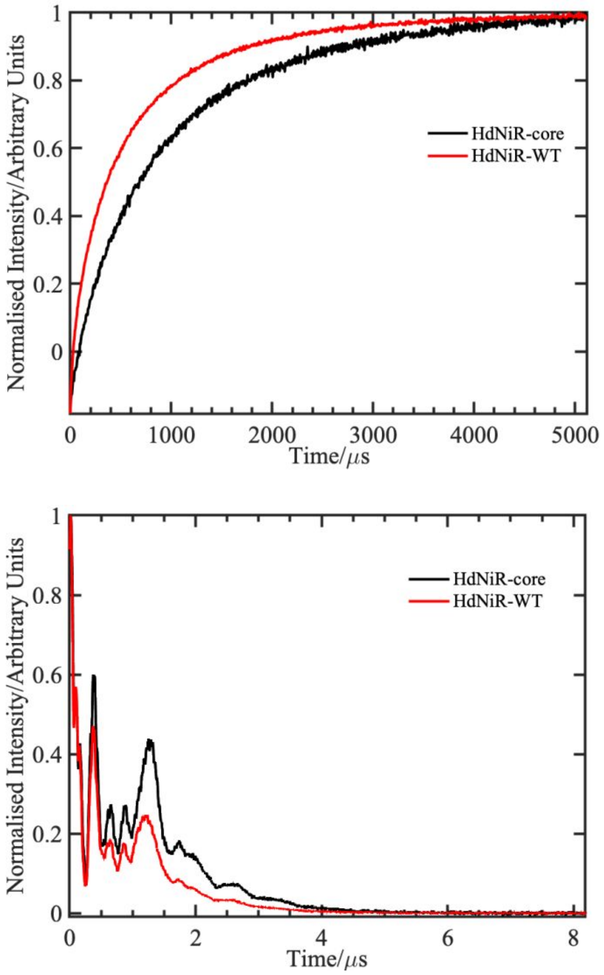

Figure S22. T_1-inversion recovery (top) and phase-memory time, T_2 (bottom) measured on the *Hd*NiR 'core' and full-length (WT) protein samples at 10 K. The magnetic fields at which T_1 and T_2 were measured are 3320 G and 3355 G for the *Hd*NiR 'core' and full-length protein samples, respectively.

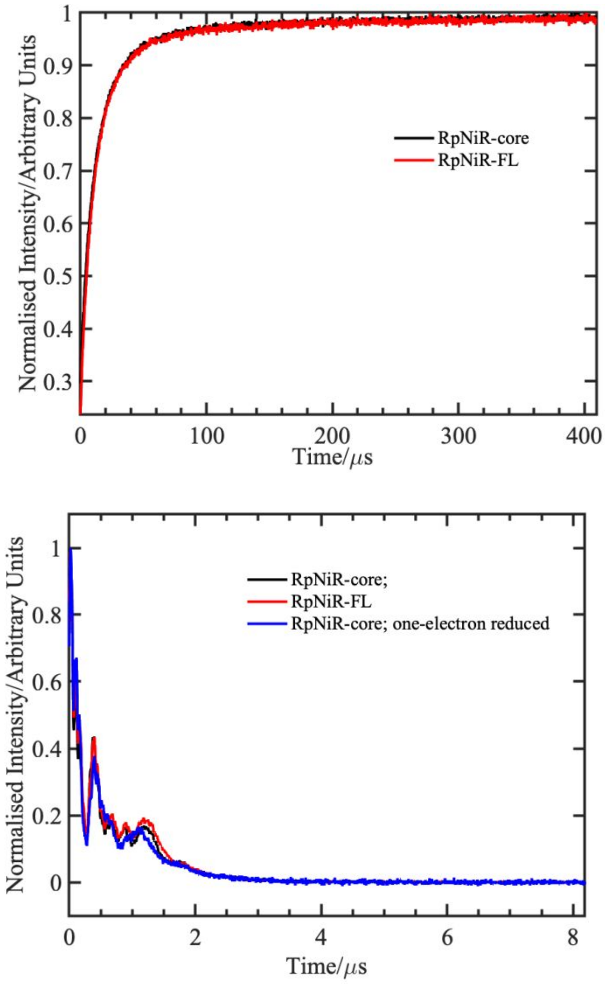

Figure S23. T_1-inversion recovery (top; 20 K) and phase-memory time, T_2 (bottom; 10 K) measured on the *Rp*NiR 'core' (black traces) and full-length (FL; red traces) protein samples at 3310 G.

Comparisons of the PELDOR results of the 'core' and full-length (WT) proteins

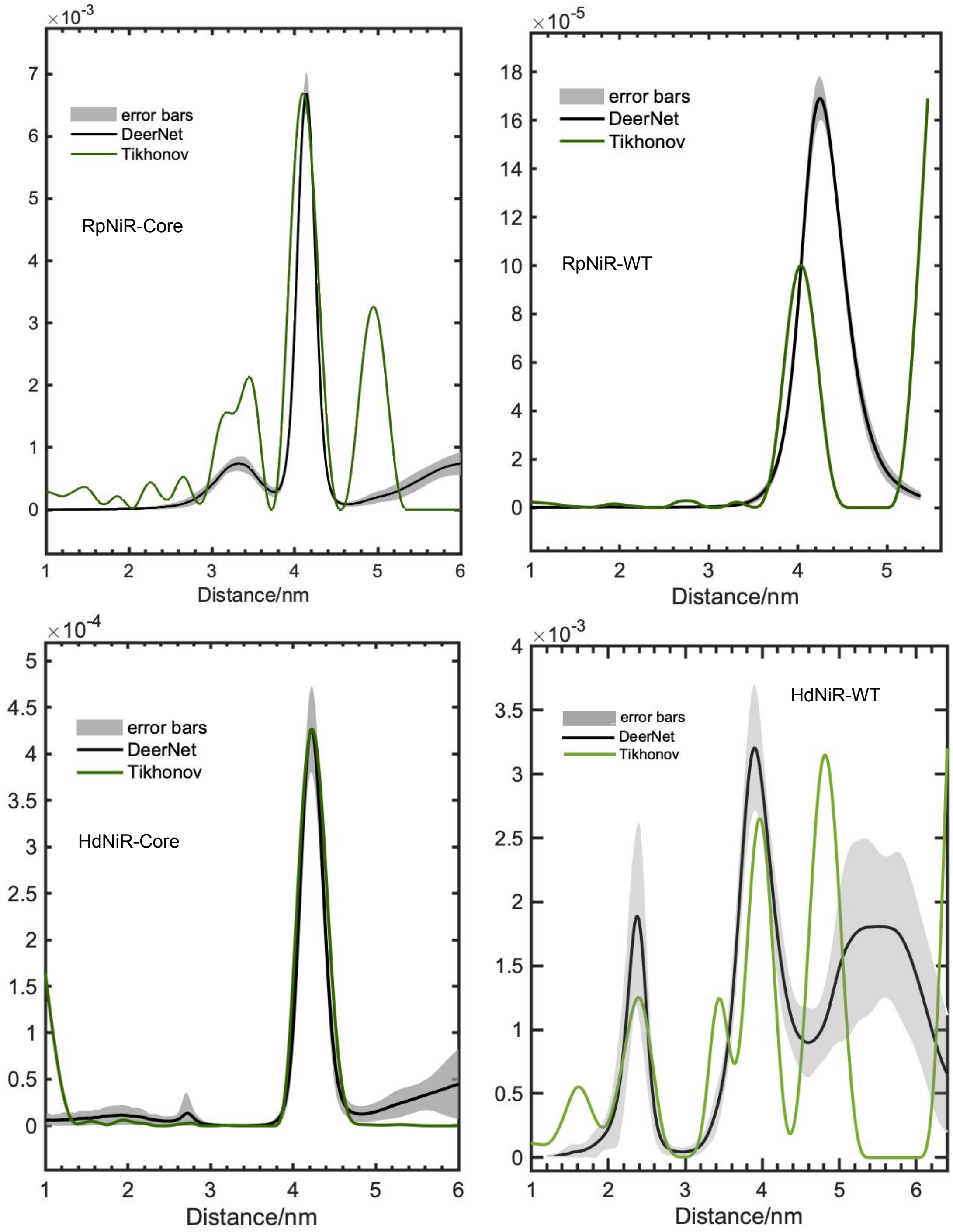

Figure S24. Comparisons of the PELDOR results of the 'core' (top and bottom left panels) and full-length (top and bottom right panels) protein samples measured at 10 K. The comparisons clearly show the conformational changes of the RpNiR-WT and a more compact geometry, whereas the HdNiR-WT PELDOR results show additional copper-copper distances consistent with the formation of the hexameric structure for the HdNiR-WT protein.

*Continuous-wave and 2-pulsed field-swept EPR spectra of the Rp-*NiR *'core' and full-length proteins:*

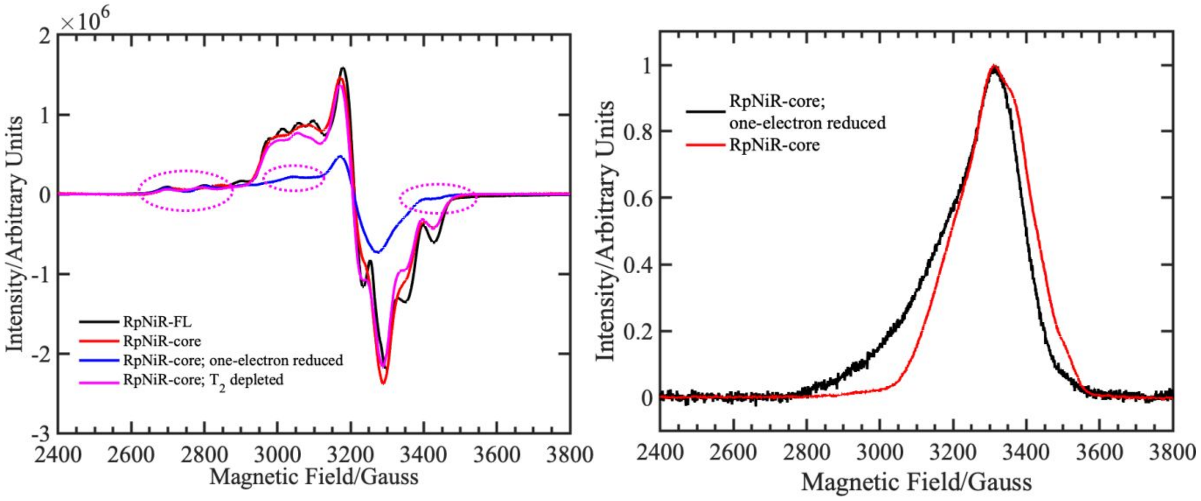

Figure S25. CW-EPR spectra of the full-length (black trace; left hand panel), 'core' regions (red trace; left hand panel), one-electron reduced (blue trace; left hand panel) and T2-depleted *Rp*NiR variants measured at 20 K. The right-hand panel shows the comparison of the echo-detected, field-swept EPR spectra of the *Rp*NiR 'core' and one-electron reduced *Rp*NiR 'core' measured at 10 K. The EPR signal due to the heme *c* cofactor (observed at 2100 G) is not shown on the left-hand panel (black trace) for comparison purposes and the reader is requested to see **Figure S3** (top) for the wide-swept EPR spectrum of the RpNiR full-length protein. Experimental conditions for the cw- and pulsed EPR are as in **Figure S3** and **Figure S7**, respectively.

Four-pulse PELDOR spectrum and distance distribution of the one-electron reduced RpNiR-“core”

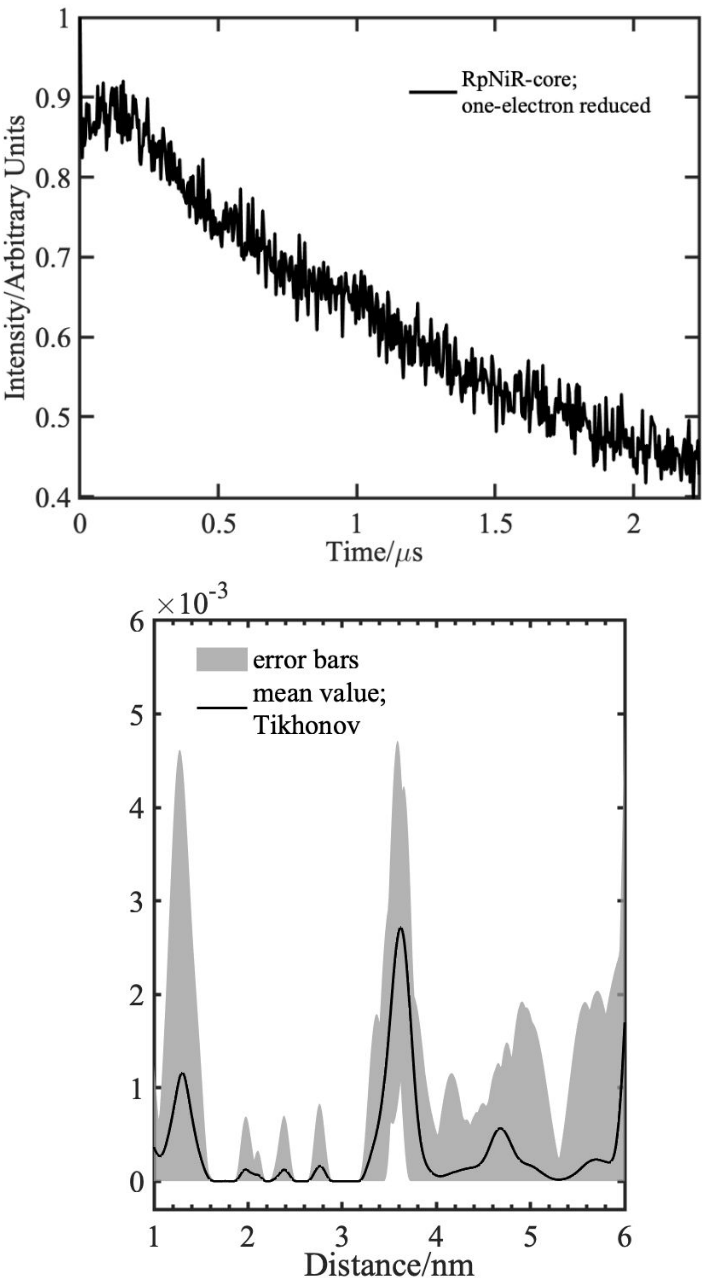

Figure S26. Four-pulse PELDOR spectrum of the one-electron reduced, *Rp*NiR-core (top panel)**.** The pulse separations and the lengths of the ‘detection/pump’ microwave pulses were as described in scheme **S1**. The spectrum was recorded at 3310 G (top) with $\upsilon_{detection}$ = 9.5757 GHz and υ_{pump} = 9.6757 GHz; the Δv ($v_{det} - v_{pump}$) was -100 MHz and the validation of a distance distribution is given in the bottom panel. The DEER trace has been averaged over “12884” scans with the shot repetition time of 3060 μs. It is noteworthy to mention that the distance distribution peak at 4.12 nm is no longer observed- which was assigned to the T1Cu-T1Cu and one of the T1Cu-T2Cu distances in the *Rp*NiR ‘core’.

Author Contributions

TMH conceived and designed experiments, prepared samples, collated, analyzed and interpreted data, wrote the manuscript, and managed the project. AII prepared samples and supported data interpretation. DC prepared samples. DH secured funds and was involved in project management. MS conceived and designed experiments, performed EPR measurements, analysed data and helped finalize the manuscript (prepare figures and edited the final manuscript). NSS secured funds and directed the project.

References

[1] J. E. O. Reilly, *Biochim. et. Biophys. Acta* **1973**, *292*, 509–515.

[2] T. M. Hedison, R. T. Shenoy, A. I. Iorgu, D. J. Heyes, K. Fisher, G. S. A. Wright, S. Hay, R. R. Eady, S. V. Antonyuk, S. S. Hasnain, et al., *ACS Catal.* **2019**, *9*, 6087–6099.

[3] S. V. Antonyuk, C. Han, R. R. Eady, S. S. Hasnain, *Nature* **2013**, *496*, 123–126.

[4] M. Nojiri, Y. Xie, T. Inoue, T. Yamamoto, H. Matsumura, K. Kataoka, Deligeer, K. Yamaguchi, Y. Kai, S. Suzuki, *Proc. Natl. Acad. Sci.* **2007**, *104*, 4315–4320.

[5] J. E. Tropea, S. Cherry, D. S. Waugh, in *High Throughput Protein Expr. Purif. Methods Protoc.* (Ed.: S.A. Doyle), Humana Press, Totowa, NJ, **2009**, pp. 297–307.

[6] G. Jeschke, V. Chechik, P. Ionita, A. Godt, H. Zimmermann, J. Banham, C. R. Timmel, D. Hilger, H. Jung, *Appl. Magn. Reson.* **2006**, *30*, 473–498.

[7] (A) S. G. Worswick, J. A. Spencer, G. Jeschke, I. Kuprov, *Sci. Adv.,* **2018**, *4*; (B) http://spindynamics.org/group/?page_id=12

[8] L. F. Ibáñez, G. Jeschke, S. Stoll, *Magn. Reson.* **2020**, *1*, 209–224.

[9] S. Stoll, A. Schweiger, *J. Magn. Reson.* **2006**, *178*, 42–55.

[10] G. E. Pake, *J. Chem. Phys.* **1948**, *16*, 327–336.

[11] Y. W. Chiang, P. P. Borbat, J. H. Freed, *J. Magn. Reson.* **2005**, *172,* 279–295.

[12] C. W. M. Kay, H. E. Mkami, R. Cammack, R. W. Evans, *J. Am. Chem. Soc.* **2007**, *129*, 4868–4869.

[13] J. H. Van Wonderen, D. N. Kostrz, C. Dennison, F. Macmillan, **2013**, *52*, 1990–1993.

[14] A. Giannoulis, C. L. Motion, M. Oranges, M. Buhl, G. M. Smith, B. E. Bode, *Chem., Phys. Chem. Phys.* **2018**, *20*, 2151–2154.

[15] T. M. Hedison, M. Shanmugam, D. J. Heyes, R. Edge, N. S. Scrutton, *Angew. Chemie - Int. Ed.* **2020**, *59*, 13936–13940.
